# ZN148 Is a Modular Synthetic Metallo-β-Lactamase Inhibitor That Reverses Carbapenem Resistance in Gram-Negative Pathogens *In Vivo*

**DOI:** 10.1128/AAC.02415-19

**Published:** 2020-05-21

**Authors:** Ørjan Samuelsen, Ove Alexander Høgmoen Åstrand, Christopher Fröhlich, Adam Heikal, Susann Skagseth, Trine Josefine Olsen Carlsen, Hanna-Kirsti S. Leiros, Annette Bayer, Christian Schnaars, Geir Kildahl-Andersen, Silje Lauksund, Sarah Finke, Sandra Huber, Tor Gjøen, Adriana Magalhaes Santos Andresen, Ole Andreas Økstad, Pål Rongved

**Affiliations:** a Norwegian National Advisory Unit on Detection of Antimicrobial Resistance, Department of Microbiology and Infection Control, University Hospital of North Norway, Tromsø, Norway; b Department of Pharmacy, UiT The Arctic University of Norway, Tromsø, Norway; c Section for Pharmaceutical Chemistry, Department of Pharmacy, University of Oslo, Oslo, Norway; d The Norwegian Structural Biology Centre (NorStruct), Department of Chemistry, UiT The Arctic University of Norway, Tromsø, Norway; e Centre for Integrative Microbial Evolution and Section for Pharmacology and Pharmaceutical Biosciences, Department of Pharmacy, University of Oslo, Oslo, Norway; f Department of Chemistry, UiT The Arctic University of Norway, Tromsø, Norway; g Department of Laboratory Medicine, Division of Diagnostic Services, University Hospital of North Norway, Tromsø, Norway

**Keywords:** antibiotic resistance, β-lactamase inhibitor, β-lactamases, carbapenem, metallo-β-lactamase

## Abstract

Carbapenem-resistant Gram-negative pathogens are a critical public health threat and there is an urgent need for new treatments. Carbapenemases (β-lactamases able to inactivate carbapenems) have been identified in both serine β-lactamase (SBL) and metallo-β-lactamase (MBL) families. The recent introduction of SBL carbapenemase inhibitors has provided alternative therapeutic options. Unfortunately, there are no approved inhibitors of MBL-mediated carbapenem-resistance and treatment options for infections caused by MBL-producing Gram-negatives are limited. Here, we present ZN148, a zinc-chelating MBL-inhibitor capable of restoring the bactericidal effect of meropenem and *in vitro* clinical susceptibility to carbapenems in >98% of a large international collection of MBL-producing clinical *Enterobacterales* strains (*n* = 234). Moreover, ZN148 was able to potentiate the effect of meropenem against NDM-1-producing Klebsiella pneumoniae in a murine neutropenic peritonitis model. ZN148 showed no inhibition of the human zinc-containing enzyme glyoxylase II at 500 μM, and no acute toxicity was observed in an *in vivo* mouse model with cumulative dosages up to 128 mg/kg. Biochemical analysis showed a time-dependent inhibition of MBLs by ZN148 and removal of zinc ions from the active site. Addition of exogenous zinc after ZN148 exposure only restored MBL activity by ∼30%, suggesting an irreversible mechanism of inhibition. Mass-spectrometry and molecular modeling indicated potential oxidation of the active site Cys221 residue. Overall, these results demonstrate the therapeutic potential of a ZN148-carbapenem combination against MBL-producing Gram-negative pathogens and that ZN148 is a highly promising MBL inhibitor that is capable of operating in a functional space not presently filled by any clinically approved compound.

## Abstract (precis)

Carbapenem-resistant Gram-negative pathogens are a critical public health threat and there is an urgent need for new treatments. Carbapenemases (β-lactamases able to inactivate carbapenems) have been identified in both serine β-lactamase (SBL) and metallo-β-lactamase (MBL) families. The recent introduction of SBL carbapenemase inhibitors has provided alternative therapeutic options. Unfortunately, there are no approved inhibitors of MBL-mediated carbapenem-resistance and treatment options for infections caused by MBL-producing Gram-negatives are limited.

## INTRODUCTION

The global increase in antimicrobial resistance is currently undermining our ability to treat bacterial infections and has become a critical public health threat worldwide. A cornerstone treatment of serious and life-threatening infections caused by multidrug-resistant (MDR) Gram-negative bacterial pathogens such as Klebsiella pneumoniae and Escherichia coli has been the carbapenem β-lactam antibiotics (e.g., meropenem) (1). The major advantage of carbapenems has been their relative stability toward β-lactamases, such as the extended-spectrum β-lactamases (ESBLs) and AmpCs, which constitute common resistance mechanisms against β-lactams (2). However, we now observe a global increase in dissemination and diversity of β-lactamases (carbapenemases) with the ability to inactivate carbapenems (3). Estimates indicate that carbapenem-resistant E. coli and K. pneumoniae caused around 3.6 million bloodstream or other serious infections globally in 2014 (4). The impact of carbapenem resistance is further illustrated in a European study where carbapenem resistance was shown to be the major contributor to the burden of infections by antibiotic-resistant bacteria in many countries (5). Moreover, a common feature of carbapenemase-producing Gram-negative bacteria is MDR, including resistance to non-β-lactam antimicrobials, resulting in severely limited treatment options (6).

β-Lactamases are divided into two main families and four classes, the serine β-lactamases (SBLs; classes A, C, and D) and the metallo-β-lactamases (MBLs; class B) (2). The main distinction between SBLs and MBLs is that SBLs possess an active site serine, whereas MBLs require the presence of zinc ions for activity. β-Lactamases with carbapenemase activity have been identified in both of these families, including SBLs, such as KPC and OXA-48-like, and the MBLs NDM, VIM, and IMP (2). The recent introduction of serine carbapenemase inhibitors such as avibactam, vaborbactam, and relebactam used in combination with β-lactams has provided treatment options against serine carbapenemase-producing Gram-negative pathogens (7, 8). Unfortunately, none of these β-lactamase inhibitors possesses inhibitory activity against MBLs. The recent Italian outbreak of NDM-producing *Enterobacteriaceae* is significant due not only to its size but also to the change in the epidemiology of carbapenem-resistant *Enterobacteriaceae* (CRE) from endemic KPC-producing CRE to NDM-producing CRE and the subsequent reduction in treatment options (9). Consequently, new treatment options for infections caused by MBL-producing Gram-negatives, including NDM-producing *Enterobacterales*, are urgently required.

Possible treatment options include cefiderocol (10) and the combination aztreonam-avibactam (11, 12). Combinations of β-lactams and β-lactam enhancers such as zidebactam (13) and nacubactam (14) have also shown promising activities. Moreover, several MBL inhibitors, including aspergillomarasmine A (15), dipicolinic acid derivatives (16), ANT431 (17), bisthiazolodines (18), and bismuth antimicrobials (19), have been reported. Recently, VNRX-5133 (taniborbactam), a dual SBL and MBL inhibitor, has shown potent activity in combination with cefepime against MBL producers (20). However, no direct MBL inhibitors are approved for clinical use (21). Here, we report the preclinical development and characterization of a synthetic and modular MBL inhibitor (ZN148) with promising *in vitro* and *in vivo* efficacy.

## RESULTS AND DISCUSSION

### Synthesis of ZN148.

**FIG 1:**
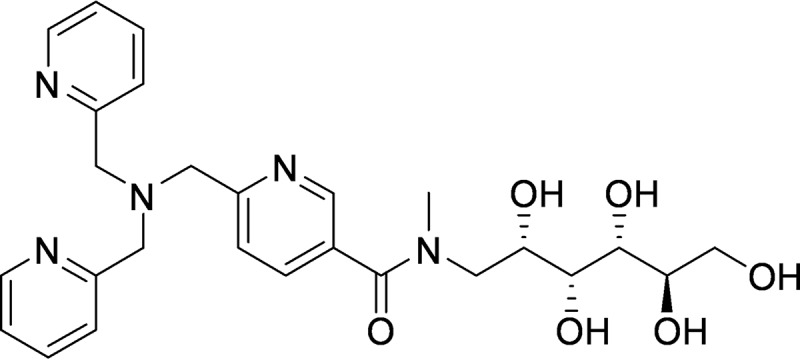
Chemical structure of ZN148.

ZN148 is a construct of the zinc chelator Tris-picolylamine (TPA) (22) covalently linked to meglumine, a hydrophilic glucosyl side chain, through an *N*-methylated amide bond, in order to lower the lipophilicity and toxicity of the chelator-conjugate (Fig. 1). TPA is a known lipophilic zinc chelator with high affinity (10^−11^ M) toward Zn^2+^ and has previously been shown to inhibit MBLs (23–26). Synthesis of ZN148 is achieved through a high-yield, three-step synthesis from commercially available building blocks (see Fig. S1 in the supplemental material), and the compound exhibits very high aqueous solubility (1.225 g/ml in phosphate-buffered saline [PBS]).

### *In vitro* activity of ZN148.

Unmodified TPA resensitized NDM-1-producing K. pneumoniae and VIM-2-producing P. aeruginosa clinical isolates toward meropenem by reducing the MIC from 32 to 64 mg/liter to 0.125 and 1 mg/liter, respectively (Table S1). However, TPA also demonstrated a high degree of toxicity against human hepatocarcinoma (HepG2) cells (50% inhibitory concentration [IC_50_] 9.8 μM). In contrast, ZN148 displayed not only a comparatively reduced toxicity against HepG2 cells (IC_50_ >100 μM), which is most likely due to its much lower cell permeability, but also retained the potentiation of meropenem activity and fully restored meropenem clinical susceptibility according to EUCAST clinical breakpoints (27) when tested at 50 μM against the same isolates (Table S1). This indicates that ZN148 is able to penetrate the Gram-negative cell wall and enter the periplasm, where the MBLs are located. Alternatively, ZN148 could lower the environmental pool of available zinc, decreasing periplasmic zinc and consequently the activity of MBLs (28). ZN148 exhibited no intrinsic antibacterial activity at concentrations up to 500 μM, confirming that the potentiation of meropenem is not due to combined antibacterial activity of the compounds.

**FIG 2:**
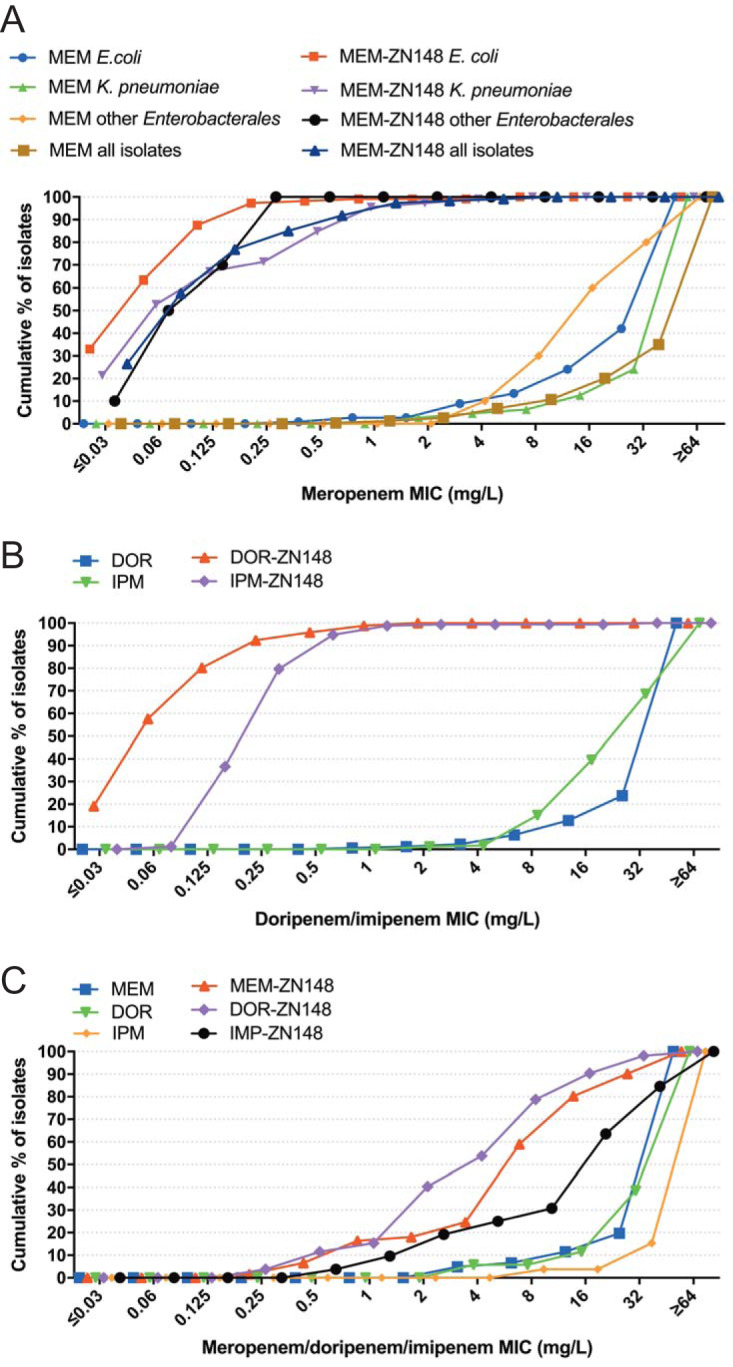
Antimicrobial activity of carbapenem-ZN148 combinations. (A) Cumulative meropenem (MEM) MIC alone or in combination with 50 μM ZN148 against MBL-producing E. coli (*n *= 112), K. pneumoniae (*n *= 112), and other *Enterobacterales* (*n *= 10) and all strains (*n *= 234) combined. (B) Cumulative doripenem (DOR) and imipenem (IPM) MIC alone or in combination with 50 μM ZN148, against MBL-producing E. coli (*n *= 87) and MBL-producing K. pneumoniae (*n *= 85). (C) Cumulative meropenem (MEM), doripenem (DOR), and imipenem (IPM) MIC alone or in combination with 50 μM ZN148 against MBL-producing P. aeruginosa. For MEM the collection included 61 strains, while for DOR and IPM the collection included 52 strains.

ZN148 at 50 μM was further tested in combination with meropenem against an extended international collection of 234 MBL-producing clinical *Enterobacterales* strains expressing variants of NDM, VIM, and IMP enzymes, as well as other non-MBL β-lactamase variants. Overall, the meropenem-ZN148 combination reduced the meropenem MIC to susceptible levels (≤2 mg/liter) in >98% of strains (MIC_90_ [meropenem] ≥64 mg/liter and MIC_90_ [meropenem-ZN148] 0.5 mg/liter) (Fig. 2A and Table S2). The geographical distribution and diversity of the strain collection shows that ZN148 is not influenced by strain background or specific MBL variants. Similarly, when tested against a subset of 173 MBL-producing E. coli and K. pneumoniae strains, ZN148 reduced the doripenem and imipenem MICs to susceptible levels in >99% of strains (Fig. 2B), indicating a potential to be used in combination with other carbapenems. Against MBL-producing P. aeruginosa (*n *= 52) and A. baumannii (*n *= 6) strains, ZN148 exhibited less potentiation of carbapenems, though still restored clinical susceptibility in 17, 15, and 25% of MBL-producing P. aeruginosa clinical strains in combination with meropenem, doripenem, or imipenem, respectively (Fig. 2C). Against NDM-1-producing class D carbapenemase-negative A. baumannii strains, ZN148 reduced the meropenem MIC >2-fold in four of six clinical strains (Table S3). The cause of the reduced effectiveness against P. aeruginosa and A. baumannii isolates is not clear but could be due to a more restricted outer membrane permeability than in *Enterobacterales* (29). This could reduce the uptake of carbapenems or ZN148 and/or a range of efflux systems which could contribute to carbapenem-resistance particularly in P. aeruginosa (30). Alternatively, if ZN148’s mode of action is through lowering the environmental availability of zinc, differential zinc uptake between *Enterobacterales* and A. baumannii/P. aeruginosa could be involved.

With a few exceptions, no potentiation of meropenem, doripenem, and imipenem was observed in strains coproducing MBLs and class D carbapenemases (Table S4). Further, no potentiation of meropenem, doripenem, or imipenem was observed for ZN148 against strains harboring only the class A carbapenemase KPC (Table S5), supporting the specificity toward MBLs.

**FIG 3:**
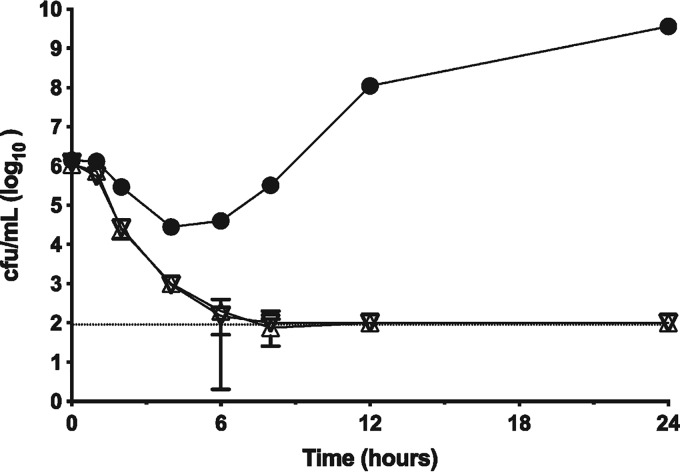
Time-kill assay. NDM-1-producing K. pneumoniae K66-45 challenged with either meropenem (MEM) alone (4 mg/liter, solid circles) or a combination of either 50 μM ZN148 plus MEM (4 mg/liter, inverted open triangles) or 100 μM ZN148 plus MEM (4 mg/liter, open triangles). Cell viability was expressed as log_10_ CFU/ml. Error bars represent the standard deviations from three independent technical replicates; the limit of detection is indicated by a dotted line.

A time-dependent cell-killing assay revealed that ZN148 restored the bactericidal activity of meropenem against an NDM-1-producing K. pneumoniae strain (Fig. 3). Meropenem alone (4 mg/liter) produced an initial bacteriostatic effect (2-log reduction in cell numbers), before eventually reaching >1 × 10^9^ CFU/ml after 24 h. In contrast, the combination of meropenem (4 mg/liter) with either 50 or 100 μM ZN148 was bactericidal, restoring a time-dependent killing mechanism. The kinetics of killing were similar for both concentrations of ZN148 tested. Cell numbers were reduced to below the limit of detection (1 × 10^2^ CFU/ml) after 8 h, and no regrowth was observed within 24 h, indicating sterilizing bactericidal activity.

Following an investigation into the frequency of resistance (FoR) using single-step selection, we observed a concentration dependent reduction in FoR for both meropenem and ZN148 (Table S6), with the FoR ranging between 10^−7^ and 10^−8^ using an NDM-1-producing K. pneumoniae strain (K66-45). Whole-genome shotgun (WGS) sequencing of stable, isolated mutants revealed mutations within the outer membrane porin OmpK36 (locus tag B5G58_03310) and a LysR type transcriptional regulator (locus tag B5G58_25480) previously associated with changes in OmpC expression in E. coli (Table S7) (31). Serial passaging of K. pneumoniae K66-45 against increasing concentrations of meropenem in the presence of 50 and 100 μM ZN148 resulted in 64× and 16× fold increases, respectively, in the MIC compared to the control culture (Fig. S3). WGS analysis of stable mutants isolated from the 100 μM ZN148 serial-passaging condition (isolated at 16-fold meropenem MIC, 2 mg/liter) also identified mutations in OmpK36 and the same LysR type transcriptional regulator as from the single-step selection (Table S8). Evolution of NDM has shown the emergence of allelic variants with enhanced zinc binding capability and increased ability to tolerate zinc starvation caused by metal chelators (32). However, no mutations were observed in the *bla*_NDM-1_ gene in either spontaneous (single-step) or serially passaged mutants emerging during exposure to ZN148, and no mutants reached clinical resistance levels. Taken together, these results demonstrated that development of resistance to ZN148 and meropenem cotreatment is unlikely to be a barrier to further development of ZN148 as an MBL inhibitor.

### ZN148 potentiates the activity of meropenem *in vivo* with no acute toxicity *in vivo*.

**FIG 4:**
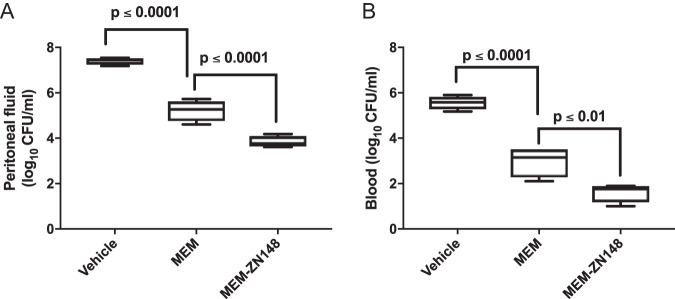
*In vivo* activity of meropenem (MEM) and MEM-ZN148 combination. Neutropenic NMRI mice were inoculated i.p. with ∼5 × 10^6^ CFU of NDM-1-producing K. pneumoniae 50752501 (MEM MIC = 64 mg/liter). Mice were treated by s.c. injection in the neck region with vehicle (PBS), MEM (33 mg/kg), or MEM (33 mg/kg) plus ZN148 (10 mg/kg). Vehicle and ZN148 were administered 1 h postinoculation, whereas MEM was administered 1.5 h postinoculation. Colony counts in blood (A) and peritoneal fluid (B) were determined at 5 h postinoculation. Four mice were included in each group. Groups were analyzed with ANOVA Dunnett´s multiple-comparison test, and *P* values of <0.05 were considered statistically significant.

In a murine neutropenic peritonitis model, subcutaneous treatment with a combination of meropenem (33 mg/kg) and ZN148 (10 mg/kg) resulted in a significantly lower CFU/ml of a meropenem-resistant NDM-1-producing K. pneumoniae strain in both peritoneal fluid (*P* < 0.0001) and blood (*P* < 0.01), compared to treatment with meropenem alone (Fig. 4). Treatment with ZN148 alone did not result in a reduction in CFU compared to vehicle treatment, corroborating the lack of intrinsic antibacterial activity observed *in vitro*. The same effect was observed for 33 and 100 mg/kg ZN148 (Fig. S2).

**FIG 5:**
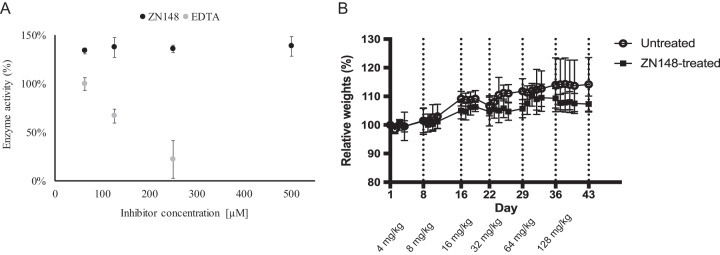
Effect of ZN148 on human glyoxylase II and *in vivo* tolerance. (A) Enzyme activity of recombinant human glyoxylase II in the presence of different concentrations of ZN148 (black circles) and EDTA (gray circles). Error bars represent standard deviations. (B) *In vivo* tolerance of ZN148 in female BALB/c mice (weekly i.p. injection) compared to untreated controls. Doses were doubled each week (4 to 128 mg/kg) in the absence of any observed weight loss or modification in behavior. Relative weights of mice in untreated (open circles) and ZN148-treated (black squares) groups are shown. The relative weight was calculated as the weight on the given day divided by the weight at day 1. The data are mean values of six mice per group, and error bars indicate the standard deviations.

Since zinc is an essential metal ion for many biological processes (33, 34), inhibitors of MBLs based on zinc chelation could potentially have off-target toxicity. To investigate this, we tested the inhibitory activity of ZN148 against the human glyoxylase II enzyme, which shares the MBL protein fold and zinc-binding properties (35). In contrast to EDTA, a strong metal chelator, ZN148 (500 μM) showed no inhibitory activity against recombinant glyoxylase II (Fig. 5A). This indicates selectivity toward bacterial MBLs and a more specific mode of action. Selectivity toward bacterial MBLs has also been shown for other zinc-chelating MBL inhibitors such as aspargillomarasmine A (15). Moreover, the inhibitory activity of ZN148 was not influenced by human serum albumin and α_1_-acid glycoprotein, indicating negligible serum protein binding (Fig. S4). In addition, no acute toxicity was observed *in vivo* in a maximum tolerated dose study using female BALB/c mice. In four groups, containing 12 animals each, after weekly doubling of single doses from 4 mg/kg up to 128 mg/kg and a 7-week cumulative administration of 252 mg/kg, no significant differences in clinical signs (hair loss or bristly hair, immobility, prostration) or loss of weight (<10% compared to initial weight) (Fig. 5B) were observed between the test and control groups.

### ZN148 irreversibly inhibits MBLs.

**FIG 6:**
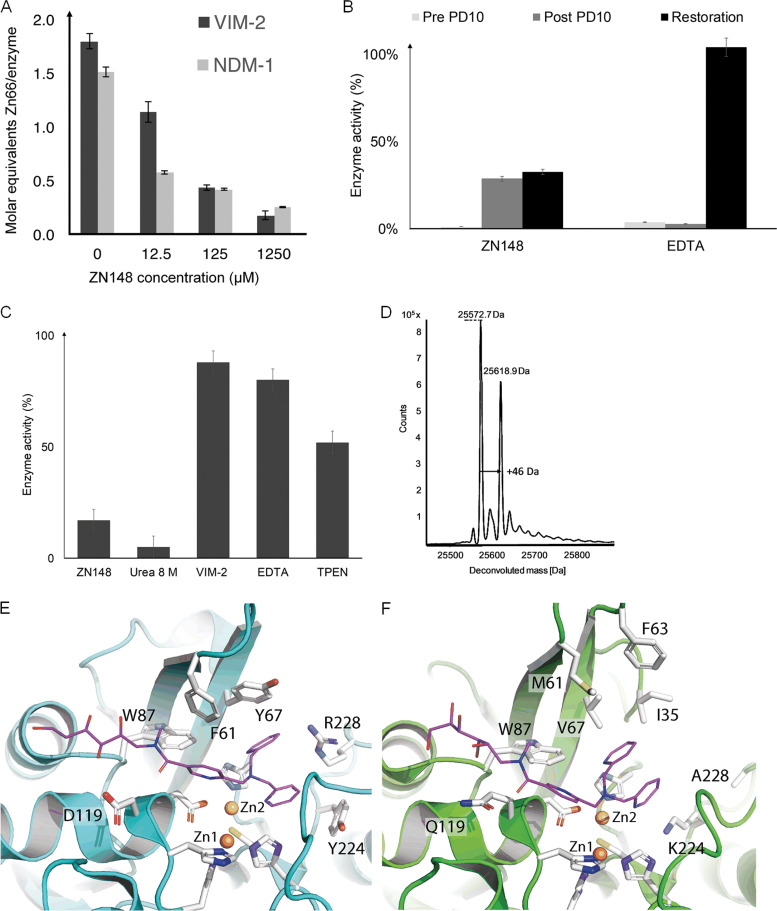
Mode of action of ZN148. (A) Zinc content determination by ICP-MS of VIM-2 and NDM-1 enzymes after preincubation with ZN148, demonstrating a removal of zinc from their active sites. (B) Preincubation of VIM-2 with ZN148 (Pre PD10), the subsequent removal of the inhibitor (Post PD10), and the restoration of enzymatic activity by adding zinc (restoration) were tested in order to describe the mode of inhibition. In contrast to EDTA, inhibition by ZN148 was irreversible and activity could not be restored. (C) Unfolding and refolding of preincubated VIM-2 with ZN148 resulted in low enzyme activities. In contrast, unfolding and refolding of VIM-2 alone or preincubated with EDTA demonstrated a refolding efficiency of >80%. (D) ESI-MS of VIM-2 preincubated with ZN148 revealed a change in mass of 46.2 ± 0.1 Da. (E and F). Molecular modeling of ZN148 (lilac) into VIM-2 (E) and NDM-1 (F). The modeling predicts aromatic interactions between ZN148 and F61, Y67, Y224, and R228 in VIM-2 (E), which are unlikely in NDM-1 due to the presence of M61, V67, K224, and A228 and where F63 is too far away for aromatic stacking (F).

The rationale behind inclusion of the TPA moiety in ZN148 was to inhibit MBLs by the removal of their active site zinc ions. Using ICP-MS for analysis of purified VIM-2 and NDM-1, we found that ZN148 removed ∼1.8 and ∼1.3 molar equivalents of zinc from VIM-2 and NDM-1, respectively (Fig. 6A). We also synthesized three ZN148 analogues, ZN222, ZN223, and ZN228, where one or several 2-pyridinyl rings were replaced with benzene rings, resulting in reduced chelator strength (23). Analysis of NDM-1 and VIM-2 enzyme kinetics revealed that the inhibitory efficiency (*k*_inact_/*K_I_*) of these compounds was lower than for ZN148 and correlated with the theoretical chelator strength (Table 1). The relative positions of the pyridine and benzene rings in the ZN148 derivatives (ZN223 and ZN228) affected the inhibitory activity (Table 1). However, none of the ZN148 analogues were able to potentiate meropenem activity toward an NDM-1-producing K. pneumoniae or a VIM-2-producing P. aeruginosa strain, i.e., the meropenem MIC was unchanged (32 mg/liter) both alone and in combination with ZN222, ZN223, or ZN228, confirming that zinc chelation is required for activity and constitutes the likely mode of inhibition for ZN148.

**TABLE 1:**
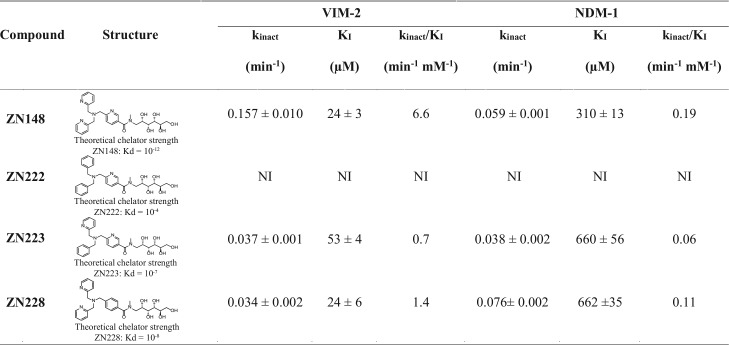
Structures and kinetic properties of ZN148, ZN222, ZN223, and ZN228 against the MBLs VIM-2 and NDM-1^*a*^

a *K_I_* represents the inhibitor concentration that leads to half-maximum inactivation, *k*_inact_ is the first-order rate constant, and *k*_inact_/*K_I_* is the catalytic efficiency. NI, no inhibition.

*In vitro* inactivation kinetics revealed that ZN148 inhibited VIM-2 (*k*_inact_/*K_I_*, 6.6 min^−1^ mM^−1^) more effectively than NDM-1 (*k*_inact_/*K_I_*, 0.19 min^−1^ mM^−1^) and showed a time-dependent inhibition of VIM-2 and NDM-1 by ZN148 (Table 1) suggestive of an irreversible mechanism of inhibition (36, 37). After incubation of VIM-2 with ZN148, the addition of exogenous zinc restored only ∼30% of the MBL activity compared to the untreated control (Fig. 6B). Unfolding and stepwise refolding of VIM-2 following incubation with ZN148 also resulted in restoration of ∼30% of the control activity (Fig. 6C). In contrast, incubation of VIM-2 with EDTA, followed by the addition of zinc, resulted in complete or nearly complete (∼80%) restoration of activity (Fig. 6B and C). Taken together, these results demonstrate a difference in the mode of inhibition for ZN148 compared to EDTA and support an irreversible mechanism of inhibition of VIM-2 by ZN148. The mechanism behind the irreversible inhibition is unclear, but several studies have postulated that chelating agents increase the susceptibility of active-site amino acids to chemical modifications, such as oxidation of Cys221 to Ocs221 (38–40). Electrospray ionization mass spectrometry (ESI-MS) analysis of purified VIM-2 incubated with ZN148 revealed an increase in mass of 46.2 ± 0.1 Da (25,572.7 ± 0.1 Da to 25,618.9 ± 0.1 Da) (Fig. 6D), potentially representative of a deprotonated cysteine sulfonic acid (47 Da). The observed mass increase indicates that removal of zinc by ZN148 renders the enzyme more susceptible to irreversible chemical modification (e.g., oxidation of Cys221) (38, 39, 41), thus preventing the restoration of enzymatic activity by refolding upon addition of exogenous zinc.

Molecular modeling of ZN148 into the active sites of VIM-2 (PDB ID 5LSC) and NDM-1 (PDB ID 4RL0) predicted favorable aromatic interactions between ZN148 and F61, Y67, and Y224 and cation-pi interactions to R228 in VIM-2 (Fig. 6E). The stacking of these aromatic side chains would likely favor greater hydrophobic and cation-pi interactions of ZN148 with the VIM-2 active site compared to the equivalent residues (M61, V67, K224, and A228) in NDM-1. ZN148 in NDM-1 would then have fewer enzyme-inhibitor interactions (Fig. 6F) and a less favorable ZN148 binding compared to VIM-2 (Table 1). This could indicate formation of a transient enzyme-Zn-inhibitor complex, followed by the removal of zinc ions from the active site, as shown for several other MBL inhibitors (42). The inactivated Zn-depleted VIM-2 enzyme demonstrated an increased mass of +46.2 ± 0.1 Da, which is likely due to the oxidation of Cys221, since oxidation to Ocs221 is observed more often in VIM (40, 43, 44) than in NDM-1 crystal structures. Second shell residues, fold, or zinc affinity might also account for an easier Cys221 oxidation in VIM-2 compared to NDM-1.

### Conclusions.

The lack of available therapies for infections caused by MBL-producing Gram-negative bacteria has prompted the World Health Organization to classify them as “priority pathogens” for research and development of new and effective treatments (45). Discovery and development of new antibiotics is fraught with difficulty and suffers from high attrition rates at early stages of development (46). The successful clinical introduction of SBL-carbapenemase inhibitors demonstrated an alternative strategy, preserving the efficacy of existing antibiotics; however, this success has not, to date, been replicated in the treatment of MBL-producing CRE.

To meet the challenge posed by MBL-producing CRE, we adopted a strategy of developing a synthetic, module zinc chelator. Taken together, the activity spectrum of ZN148 in combination with carbapenems, *in vivo* efficacy, and limited *in vivo* toxicity demonstrate that ZN148 has the potential to enter preclinical studies for further development. Furthermore, as a modular and easy-to-synthesize MBL inhibitor that restores the activity of carbapenems toward a range of clinically important Gram-negative MDR pathogens, ZN148 is well placed to enter the clinical development pipeline along with other promising recently approved molecules (47) in meeting the urgent, therapeutic challenge of global importance.

## MATERIALS AND METHODS

The chemical synthesis of ZN148 and analogues ZN222, ZN223, and ZN228 is described in the supplemental material and in Fig. S1.

### Solubility measurements of ZN148 in PBS.

The solubility of ZN148 was evaluated in PBS buffer at pH 7.4. Increasing amounts of ZN148 was dissolved in 1.0 ml at room temperature by gentle swirling of the solution in a glass vial. A total of 1.225 g of ZN148 was successfully dissolved in 1.0 ml of PBS buffer, giving a total volume of 1.8 ml. This solution was stable at 4°C for more than 6 weeks without precipitation.

### Antibacterial susceptibility testing and time-kill experiments.

MICs of carbapenems alone and in combination with inhibitors were determined by broth microdilution according to the Clinical and Laboratory Standards Institute (CLSI) guidelines (48) using either premade plates (TREK Diagnostic Systems/Thermo Fisher Scientific, East Grinstead, United Kingdom) with meropenem or in-house-prepared plates with meropenem, imipenem, and doripenem. ZN148 and other inhibitors were tested at a fixed concentration of 50 μM. Cation-adjusted Mueller-Hinton medium (TREK Diagnostic Systems/Thermo Fisher Scientific; Becton Dickinson, Franklin Lakes, NJ) were used as growth medium. The plates were incubated for 20 h at 37°C. ZN148, ZN222, ZN223, ZN228, and TPA were initially tested in combination with meropenem using two indicator strains: NDM-1-producing K. pneumoniae (49, 50) and VIM-2-producing P. aeruginosa (51). The activity of ZN148 were subsequently tested against two extended strain collections: (i) in combination with meropenem against MBL-producing *Enterobacterales* (*n *= 62), MBL-producing P. aeruginosa (*n *= 9) and MBL- and class D carbapenemase-coproducing *Enterobacterales* (*n *= 5) from the strain collection at the Norwegian National Advisory Unit on Detection of Antimicrobial Resistance and (ii) in combination with meropenem, imipenem, and doripenem against globally collected MBL-producing E. coli (*n *= 87), K. pneumoniae (*n *= 85), P. aeruginosa (*n *= 52), and A. baumannii (*n *= 6), MBL- and class A/D carbapenemase-coproducing E. coli (*n *= 11), K. pneumoniae (*n *= 15) and A. baumannii (*n *= 7) at IHMA Europe, Sàrl, Switzerland. E. coli ATCC 25922 and P. aeruginosa ATCC 27853 were used as quality control strains. The MBL inhibitors were also tested for intrinsic antibacterial activity against the strains.

To determine the kinetics of killing of the meropenem-ZN148 combination, NDM-1-producing K. pneumoniae K66-45 (1 × 10^6^ CFU/ml) in cation-adjusted Mueller-Hinton broth II (Sigma-Aldrich, St. Louis, MO) was treated with either 4 mg/liter meropenem or a combination of 4 mg/liter meropenem plus 50 or 100 μM ZN148 and grown at 37°C with shaking (180 rpm). The cell number was determined over the course of 24 h by using a modified Miles-Misra method (52). Briefly, 10-ml serial dilutions of culture in sterile PBS were spotted and dried on square (10 by 10 cm) Luria-Bertani agar (Oxoid, Basingstoke, UK) plates at room temperature before incubation at 37°C. The limit of detection was set as two colonies in 10 ml of undiluted culture, representing 100 CFU/ml.

### *In vivo* efficacy study.

The *in vivo* efficacy of meropenem (Mylan; Mylan Hospitals, Asker, Norway) in combination with ZN148 were evaluated in a murine neutropenic peritonitis model. Female NRMI mice (Taconic Biosciences, Lille Skensved, Denmark) were rendered neutropenic by intraperitoneal (i.p.) injection with cyclophosphamide (Baxter, Søborg, Denmark) at 4 days (200 mg/kg) and 1 day (100 mg/kg) prior to inoculation. Mice were inoculated i.p. with ∼5 × 10^6^ CFU of NDM-1-producing K. pneumoniae 50752501. At 1 h postinoculation, the mice were injected subcutaneously (s.c.) in the neck region with ZN148 corresponding to 10, 33, or 100 mg/kg or vehicle. After 30 min, the mice were injected s.c. with 33 mg/kg meropenem or vehicle. Four mice were included for each treatment regime. At 5 h postinoculation, the mice were anesthetized. Blood was collected from axillary cutdown, and the mice were sacrificed. Subsequently, 2 ml of sterile saline was injected i.p., and the intraperitoneal fluid was sampled. Blood and intraperitoneal fluid were serially diluted in 0.9% NaCl and plated on blood agar plates with 5% horse blood (Statens Serum Institut, Copenhagen, Denmark) for CFU determination. Groups were analyzed with analysis of variance (ANOVA) Dunnett’s multiple-comparison test in Prism 7.04 (GraphPad Software, Inc.). *P* values of <0.05 were considered statistically significant. All animal experiments were approved by the National Committee of Animal Ethics, Denmark (permission 2014-15-0201-00171), and adhered to the standards of EU Directive 2010/63/EU.

### Single-step selection of *K. pneumoniae* K66-45 for growth on meropenem and ZN148 in combination.

In order to determine the frequency of resistance to the combination of meropenem and ZN148, a modified single-step selection experiment was carried out as previously descrwithibed (53). Briefly, NDM-1-producing K. pneumoniae K66-45 was grown from a single colony to approximately 10^9^ CFU/ml and plated on cation-adjusted Mueller-Hinton broth II (Becton Dickinson) agar containing 30, 50, 100, or 200 μM ZN148 and 0.5, 1, 2, 4, or 8 mg/liter meropenem in combination. Colonies were counted after overnight incubation at 37°C. The concentrations of meropenem chosen ranged from 4× to 64× MIC (MIC values in the absence of ZN148) and correlated with EUCAST clinical breakpoints for *Enterobacterales*, which define ≤2 mg/liter as sensitive and >8 mg/liter as resistant to meropenem (18).

### Serial passage of *K. pneumoniae* K66-45.

K. pneumoniae K66-45 was passaged in a 96-well microtiter plate with a consistent concentration of ZN148 and increasing concentrations of meropenem. To start the passaging, cation-adjusted Mueller-Hinton broth II medium (Sigma-Aldrich) containing 0.0625 mg/liter (0.5× MIC) and 50 or 100 μM ZN148 was inoculated with 1% of overnight culture, which was grown without selection. Six biological replicates were passaged in parallel, and from each of the six overnight cultures four wells were inoculated, resulting in 24 subcultures for each ZN148 concentration. After 24 h of incubation at 37°C and shaking at 180 rpm, the wells were visually checked for growth. Passaging was continued with all subcultures showing growth by transferring 1 μl of subculture to 100 μl of fresh medium with 0.125 mg/liter meropenem (1× MIC) and 50 or 100 μM ZN148. This process was continued by increasing the meropenem concentration by 2-fold every 24 h until none of the subcultures showed growth.

### DNA preparation and *de novo* whole-genome sequencing of evolved clones from single-step selection and serial passaging experiments.

Preparation of genomic DNA from NDM-1-producing K. pneumoniae K66-45 was carried out with a MO BIO DNeasy UltraClean microbial kit (Qiagen) as previously described (49). Whole-genome shotgun (WGS) sequencing was carried out at either the Norwegian Sequencing Centre (Oslo, Norway) or Novogene (Beijing, China) using the Illumina HiSeq platform. Variations and single nucleotide polymorphisms in mutant WGS sequences with greater than 10× coverage were identified by mapping Illumina reads to the reference genome (GenBank accession numbers CP020901 to CP020905) (49) using Bowtie2 (54) within Geneious version 11.1.5 (55) and using the inbuilt Geneious variant/SNP finder command.

### *In vitro* toxicity.

The human hepatoblastoma HepG2 cell line (HB-8065; ATCC, Manassas, VA) was cultured in DMEM-GlutaMAX (5.5 mM glucose; Thermo Fisher) supplemented with 10% fetal bovine serum (Sigma-Aldrich), streptomycin (100 μg/ml; Gibco/Thermo Fisher), and penicillin (100 U/ml; Gibco/Thermo Fisher) at 37°C in 5% CO_2_. For viability assays, cells were seeded in white 96-well Nunclon plates (Sigma-Aldrich) at a density of 20,000 cells/well and left overnight to adhere. ZN148 and TPA (Sigma-Aldrich, Darmstadt, Germany) dissolved in dimethyl sulfoxide (DMSO; (Sigma-Aldrich) were added to the white 96-well plates containing 20,000 HepG2 cells/well at concentrations ranging from 1 to 1 × 10^−3 ^mM (a DMSO concentration kept below 1%), followed by incubation for 24 h at 37°C in 5% CO_2_. After 24 h, alamarBlue cell viability reagent (Thermo Fisher, Carlsbad, CA) was added (10% final concentration), followed by incubation for 4 h at 37°C. The proportion of viable cells in each well was measured in a fluorescence plate reader (Clariostar; BMGlabtech, Germany) at excitation 550 nm/emission 603 nm (56). Data from eight replicates were fitted by nonlinear regression to determine IC_50_ values using Prism (GraphPad Software, Inc.).

### *In vivo* tolerance.

*In vivo* tolerance of ZN148 was performed by Antineo (Lyon, France). Female BALB/c mice (4 weeks old, approximately 20 g; Charles River, L’Arbresle, France) were acclimatized 4 days in the animal facility before the initiation of experiments. Groups of six mice were either untreated or treated with 200 μl of ZN148 (0.4 to 12.8 g/liter) i.p. once a week. Doses were doubled each week (4 to 128 mg/kg) in the absence of any observed weight loss or modification in behavior. Individual weights were monitored 4 days a week. The relative weights were calculated as ratios between the weight of the day and the weight at the initiation of the experiment. Animals were also followed for macroscopic modifications in behavior. The protocol for experiments in mice was approved by the University of Lyon Animal Ethics Committee (Comité d’Ethique en Expérimentation Animale de l’Université Claude Bernard [Lyon, France], authorization number DR2015-09).

### Protein binding.

Protein binding of ZN148 were investigated using a Transil^XL^ plasma protein binding kit (Sovicell, Leipzig, Germany) containing human serum albumin and α_1_-acid glycoprotein in a ratio of 24:1. Next, 500 μM ZN148 in 50 mM HEPES buffer (pH 7.5) was added to different concentrations (0 to 140 μM) of the protein mixture, followed by incubation for 12 min at room temperature with shaking at 1,200 rpm. After incubation, the suspensions were centrifuged for 5 min on a Minifuge (Starlab, Milton Keynes, UK), and the supernatant was diluted 1:10 in 50 mM HEPES buffer (pH 7.5) supplemented with 1 μM ZnSO_4_ (Sigma-Aldrich). VIM-2 was added at final concentration of 1 nM, and the solution was incubated for 10 and 30 min at 25°C. The enzyme activity was measured by adding nitrocefin (Merck, Darmstadt, Germany) at a final concentration of 50 μM and measured at 482 nm at 25°C in a SpectraMax Plus plate reader (Molecular Devices). The use of buffer alone was included as a control.

### Interaction with human glyoxylase II.

Recombinant human glyoxylase II (rHGly II; R&D Systems) was incubated with or without ZN148 (final concentrations of 125, 250, and 500 μM) for 10 and 30 min at 25°C in 50 mM Tris buffer (Merck) supplemented with 250 mM NaCl (pH 7.5). Then, 50 μl of substrate mixture containing 2 mM *S*-lactoylglutathione and 400 μM 5,5′-dithiobis(2-nitrobenzoic acid) (Sigma-Aldrich) was mixed with 50 μl of the preincubated rHGly II at a final enzyme concentration of 0.2 ng/μl. The initial enzymatic velocity was measured at 405 nm in 96-well plates (Thermo Fisher Scientific, Roskilde, Denmark) at 25°C in a SpectraMax Plus plate reader (Molecular Devices). EDTA (Merck) was included as a control.

### Time-dependent inactivation kinetics.

Protein expression and purification of NDM-1 and VIM-2 were performed as previously described (36, 43, 57). Stock solutions of purified NDM-1 and VIM-2 were prepared in 50 mM HEPES buffer (pH 7.5; Merck). Inhibition of NDM-1 and VIM-2 were determined for ZN148 and the analogues ZN222, ZN223, and ZN228 at different concentrations of inhibitor after preincubation times of 2, 8, 15, 25, and 32 min in 50 mM HEPES buffer (pH 7.5) supplemented with 1 μM ZnSO_4_ (Sigma-Aldrich) and bovine serum albumin (Sigma-Aldrich; final concentration, 2 μg/ml) at 25°C. Concentrations of 1 nM VIM-2 and 30 nM NDM-1 were used, and the reaction was initiated by the addition of 30 μM nitrocefin (Merck) for VIM-2 or 100 μM imipenem (Sigma-Aldrich) for NDM-1. The reaction was measured at 482 nm (VIM-2) or 300 nM (NDM-1) in either standard 96-well plates (Thermo Fisher Scientific) for VIM-2 or UV-transparent 96-well plates (Corning, Kennebunk, ME) for NDM-1 at 25°C in a SpectraMax Plus plate reader (Molecular Devices). All of the enzyme and substrate concentrations indicated are the final concentrations in the assay. The enzyme activity as a percentage was calculated based on the initial velocity and compared to the control without inhibitor compound. All tests were performed at least in duplicates.

The observed rate constant (*k*_obs_) per inhibitor concentration was calculated from the slope of a semilog plot of the enzyme activity in percent versus preincubation time. The individual values of *k*_obs_ were plotted against the inhibitor concentration and saturation kinetics were fitted into equation 1 by using GraphPad Prism 4 based on the following model:

$$\text{E-Zn}+\text{I}\overset{K_{I}}{↔}\text{E-Zn-I}\overset{k_{\text{inact}}}{\to }\text{E}+\text{Zn-I and E}*\text{-Zn-I}$$

where K_I_ represents the inhibitor concentration that leads to an half-maximum inactivation of the enzyme, *k*_inact_ is the first-order rate constant, E-Zn^2+^ is the holoenzyme, I is the inhibitor, E-Zn-I is the enzyme-Zn-inhibitor ternary complex, E is the inactive Zn-depleted enzyme, Zn-I is the zinc-inhibitor complex, and E*-Zn-I is the inactive enzyme-Zn-inhibitor ternary complex (39).

(1) $$k_{\text{obs}}=\frac{k_{\text{inact}}[\text{I}]}{\frac{\left[\text{S}\right]}{K_{I}}+[\text{I}]}$$

By fitting these values, the irreversible kinetic parameters maximum inactivation rate (*k*_inact_) and the inhibitor concentration that produces half-maximal rate of inactivation (*K_I_*) were obtained. Finally, the inhibitors were characterized by calculating *k*_inact_/*K_I_*. Where no saturation curve could be observed, *K_I_* and *k*_inact_ were determined from the linear part of plot 1/*k*_obs_ versus 1/[I].

### Zinc^66^ determination by ICP-MS.

Inductively coupled plasma mass spectrometry (ICP-MS) was used to investigate the chelating property of ZN148 by measuring the zinc concentration (Zn^66^) after incubation of VIM-2 with or without ZN148 in zinc depleted 50 mM HEPES buffer (pH 7.5; Chelex HEPES buffer). The Chelex buffer was prepared by stirring 2 g of Chelex resin (Bio-Rad, Hercules, CA) in 100 ml of 50 mM HEPES buffer (pH 7.5). The resin was subsequently removed by sterile filtration (Merck Millipore, 0.22 μm). VIM-2 (10 g/liter) was diluted to 12.5 mg/liter and mixed with ZN148 at different final concentrations (0, 12.5, 125, and 1250 μM) in Chelex HEPES buffer. All solutions were allowed to incubate for 30 min on ice and subsequently concentrated by centrifugation (at 4,000 × *g*, 4°C for 25 min) to a volume of 250 μl using centrifugal molecular cutoff filters (Merck Millipore, 10,000 Da). The residual inhibitor within these samples was diluted by factor of 5,000 using the same type molecular cutoff filters and 50 mM Chelex HEPES buffer (pH 7.5). The VIM-2 protein was concentrated to 0.2 g/liter, followed by a 1/16 dilution with 750 μl of a diluent mixture containing Rh^103^ (Inorganic Ventures, Christiansburg, VA) as an internal standard. The diluent mixture consisted of Milli-Q water (Millipore/Merck KGaA, Darmstadt, Germany) with 2 μg/Rh^103^, 2.5% (vol/vol) ammonia solution (Honeywell Fluka, Bucharest, Romania), 0.08% (vol/vol) Triton X-100 (Sigma/Merck KGaA, Darmstadt, Germany), 10% (vol/vol) isopropanol (Honeywell Fluka, Bucharest, Romania), and 0.25 μg/liter Au (Inorganic Ventures, Christiansburg, VA) as stabilizer. The samples were introduced to the nebulizer (N2 gas flow, 1.03 ml/min) by an ESI-Fast SC2DX autosampler with a sample flow rate of 3 rpm and further into the NexION 300D ICP-MS system (Perkin-Elmer, Waltham, MA). Per inhibitor concentration, at least three biological replicates were performed and injected and measured in triplicates. For the MS analysis the kinetic energy discrimination mode with a helium flow rate of 5.7 ml/min, 20 sweeps per reading, and a dwell time of 100 ms/atomic mass unit (AMU) for Zn^66^ and 50 ms/AMU for Rh^103^ were applied. The measurements were performed with following instrumental settings: rf power (1,600 W), plasma gas flow (18 ml/min Ar), auxiliary gas flow (1.2 ml/min N2), RPQ voltage (0.25 V), and integration time (2,000 ms). All zinc concentrations were obtained by the internal standard method, followed by a blank subtraction using the NexION software version 1.5 (Perkin-Elmer). The zinc concentration within the samples was determined based on an external calibration curve (0, 290, 580, 1,160, and 2,320 μg/liter). As controls, VIM-2 protein without inhibitor, buffer control, and diluent blanks for the control of instrumental carryover and one sample for quality control of the measurements (580 μg/liter standard) were included.

### LC-ESI-MS.

VIM-2 (8 μM final concentration) was incubated together without or with 5 mM ZN148 on ice for 30 min. Incubation was carried out in Chelex HEPES buffer (pH 7.5), and the inhibitors were diluted in Milli-Q water (Millipore/Merck) by a factor of 1,000,000 using centrifugal filters (10,000 Da; Merck). Liquid chromatography electrospray ionization Q-TOF mass spectrometry (LC-ESI-MS; Agilent Technologies, Santa Clara, CA) was performed in positive-ion mode using 0.1% formic acid in Milli-Q water and acetonitrile (VWR, Radnor, PA) at 0.4 ml/min. Spectra deconvolution was performed by using BioConfirm and MassHunter qualitative analysis (Agilent Technologies).

### Reversibility and zinc restoration of enzyme activity.

For reversibility of enzyme activity after incubation with ZN148, 3 ml of VIM-2 or NDM-1 was diluted with or without ZN148 in Chelex HEPES buffer (pH 7.5) and subsequently incubated for 1 h on ice. Then, 2.5 ml of each solution was loaded onto an equilibrated PD-10 column (GE Healthcare, Pittsburgh, PA), washed with 1 ml, and eluted with 1.5 ml of Chelex HEPES buffer (pH 7.5), respectively. Samples spiked and not spiked with inhibitor were diluted to a final enzyme concentration of 1 nM VIM-2 and 3 nM NDM-1. The initial reaction velocity was determined by adding nitrocefin (Merck) for VIM-2 or imipenem (Sigma-Aldrich) for NDM-1 at final concentrations of 50 and 100 μM, respectively. The initial enzyme reactions were recorded at 25°C. Inhibitor and enzyme alone were included as controls. All measurements were performed at least in duplicates. For enzyme restorability, pre- and postcolumn samples were supplemented with ZnSO_4_ (Sigma-Aldrich) to a final concentration of 100 μM and allowed to incubate for 5 min at 25°C. The initial enzyme velocity was then determined as described above.

### Unfolding and refolding of VIM-2.

VIM-2 (0.4 μM) was preincubated on ice for 30 min with or without ZN148. Complete protein unfolding was archived in 50 mM HEPES buffer (pH 7.5) supplemented with 1 μM ZnSO_4_ (Sigma-Aldrich) and 8 M urea (Merck, Darmstadt, Germany). Refolding was achieved by a stepwise buffer exchange using centrifugal cutoff filters (10 kDa; Merck) and decreasing concentrations of urea (0, 2, 4, and 6 M in 50 mM HEPES buffer [pH 7.5]). VIM-2 was diluted to a final concentration of 1 nM, and the enzyme activity was measured by adding nitrocefin (Merck) to a final concentration of 50 μM.

### Modeling of ZN148 into VIM-2 and NDM-1.

ZN148 was modeled into VIM-2 and NDM-1 based on X-ray crystallography data of VIM-7 cocrystallized with ZN222 (C. Froelich and H-K. S. Leiros et al. [unpublished data]), the protein-inhibitor/substrate complex structures of VIM-2 with triazolylthioacetamide (PDB ID 5LSC) and NDM-1 bound to cefuroxime (PDB ID 4RL0) by using Phenix version 1.12 (58). The 2-pyridinyl rings in ZN148 were first placed at the same sites as for ZN222, and then the linker of the inhibitor exited the active site toward W87, N233, and residue 119 similar to the inhibitor triazolylthioacetamide in VIM-2 (PDB ID 5LSC).

## Supplementary Material

Supplemental file 1
