# Supplementary material for: ZN148 Is a Modular Synthetic Metallo-β-Lactamase Inhibitor That Reverses Carbapenem Resistance in Gram-Negative Pathogens In Vivo
Source: Antimicrob Agents Chemother. 2020 May 21;64(6):e02415-19. doi: 10.1128/AAC.02415-19 (PMC7269481; doi:10.1128/AAC.02415-19)
Supplement: Supplemental file 1 [file AAC.02415-19-s0001.pdf]

Supplemental Material for

ZN148 – a modular synthetic metallo-β-lactamase inhibitor reverses carbapenem-resistance in Gram-negative pathogens *in vivo*

Authors: Ørjan Samuelsen[a,b,1], Ove Alexander Høgmoen Åstrand[c], Christopher Fröhlich[a,d], Adam Heikal[e], Susann Skagseth[d], Trine Josefine Olsen Carlsen[d], Hanna-Kirsti S. Leiros[d], Annette Bayer[f], Christian Schnaars[c], Geir Kildahl-Andersen[c], Silje Lauksund[a,b], Sarah Finke[e], Sandra Huber[g], Tor Gjøen[e], Adriana Magalhaes Santos Andresen[e], Ole Andreas Økstad[e], Pål Rongved[c,1]

Author Affiliations: [a]Norwegian National Advisory Unit on Detection of Antimicrobial Resistance, Department of Microbiology and Infection Control, University Hospital of North Norway, Tromsø, Norway. [b]Department of Pharmacy, UiT The Arctic University of Norway, Tromsø, Norway. [c]Section for Pharmaceutical Chemistry, Department of Pharmacy, University of Oslo, Oslo, Norway. [d]The Norwegian Structural Biology Centre (NorStruct), UiT The Arctic University of Norway, Tromsø, Norway. [e]Centre for Integrative Microbial Evolution (CIME) and Section for Pharmacology and Pharmaceutical Biosciences, Department of Pharmacy, University of Oslo, Blindern, Oslo, Norway. [f]Department of Chemistry, UiT The Arctic University of Norway, Tromsø, Norway. [g]Department of Laboratory Medicine, Division of Diagnostic Services, University Hospital of North Norway, Tromsø, Norway.

[1] To whom correspondence may be addressed: E-mail: orjan.samuelsen@unn.no or pal.rongved@farmasi.uio.no

SUPPLEMENTAL MATERIALS AND METHODS

The general design and chemical synthesis of ZN148 and analogues ZN222, ZN223 and ZN228 is described below and in Fig. S1.

General experimental procedures:

All reagents and solvents were of analytical grade and were used as received, without further purification. *N,N*-Diisopropylethylamine (DIPEA) and di-(2-picolyl)amine (DPA) were purchased from TCI Europe, Zwijndrecht, Belgium. methyl 6-(bromomethyl)nicotinate was purchased from BOC Sciences, Shirley NY, USA. Deuterated solvents for NMR were purchased from Fluorochem, Hadfield, UK. All other reagents and solvents were purchased from Sigma Aldrich/Merck. ^{1}H spectra were recorded with Bruker AVII 400, AVIII 400, DPX 300 or AVI 600 Fourier transform spectrometers, using an internal deuterium lock, operating at 400 MHz, 400 MHz, 300 MHz or 600 MHz respectively. ^{13}C NMR spectra were recorded with Bruker AVII 400, AVIII 400, DPX 300 or AVI 600 Fourier transform spectrometers, using an internal deuterium lock, operating at 100 MHz, 100 MHz, 75 MHz or 150 MHz respectively. All spectra were recorded at 25 °C. Chemical shifts are reported in parts per million (ppm) relative to residual protons or carbons of deuterated solvent (δ = 2.50 ppm for ^{1}H NMR and δ = 39.52 ppm for ^{13}C NMR for DMSO-*d*6, δ = 7.26 ppm for ^{1}H NMR and δ = 77.16 ppm for ^{13}C NMR for $CDCl_3$, δ = 3.31 ppm for ^{1}H NMR and δ = 49.00 ppm for ^{13}C NMR for CD_3OD). Mass spectra were recorded at 70 eV on a Waters Prospec Q or Micromass QTOF 2W spectrometer using ESI or APCI as the method of ionization. High resolution mass spectra were recorded at 70 eV on a Waters Prospec Q or Micromass QTOF 2W spectrometer using ESI or APCI as the method of ionization. TLC analyses were carried out using Merck Aluminum Oxide 60 F_{256} or Merck Silica gel 60 RP-18 pates visualized by UV light. Agilent Bondesil C18-OH or Versaflash C18 column material supplied by SigmaAldrich were used as stationary phases for reverse phase dry vacuum chromatography. The yields reported are of isolated material and are uncorrected for purity.

Chemical synthesis:

Methyl 6-((bis(pyridin-2-ylmethyl)amino)methyl)nicotinate (**ZN145**). The target compound was prepared according to a published literature procedure (1). Methyl 6-(bromomethyl)nicotinate (12.979 g, 56.4 mmol, 1.0 eq.) was suspended in 400 mL tetrahydrofuran (THF) at room temperature. Dipicolylamine (13.49 g, 12.15 mL, 67.7 mmol, 1.2 eq.) and DIPEA (16.7 mL, 95.89 mmol, 1.7 eq.) were then added and the reaction mixture was stirred at room temperature for 16 h, then concentrated under reduced pressure to approximately 200 mL. The suspension was filtered through a paper filter, the solid washed with THF (2x50 mL) and the obtained solution concentrated under reduced pressure. The residual dark brown oil was dissolved in 100 mL diethyl ether, filtered through a plug of celite and stored in the freezer to obtain 6-((bis(pyridin-2-ylmethyl)amino)methyl)nicotinate (11.14 g, 31.9 mmol, 56%) as a pale yellow solid. The obtained 1H-NMR data was in accordance with the published data. 1H NMR (300 MHz, $CDCl_3$) δ 9.11 (dd, J = 2.1, 0.7 Hz, 1H), 8.53 (ddd, J = 4.8, 1.6, 0.8 Hz, 2H), 8.24 (dd, J = 8.2, 2.2 Hz, 1H), 7.72 –

7.60 (m, 3H), 7.53 (d, J = 7.8 Hz, 2H), 7.14 (ddd, J = 7.3, 4.9, 1.1 Hz, 2H), 3.95 (s, 2H), 3.92 (s, 3H), 3.89 (s, 4H). The spectroscopic data are in accordance with published data (1).

6-((bis(pyridin-2-ylmethyl)amino)methyl)nicotinic acid (**ZN146**). Methyl 6-((bis(pyridin-2-ylmethyl)amino)methyl)nicotinate (**ZN145**) (1.7335 g, 4.97 mmol, 1.0 eq.) was dissolved in 20 mL THF and cooled in an ice bath. A solution of LiOH hydrate (626 mg, 14.92 mmol, 3.0 eq.) in 20 mL dest. H_2O was added and the solution stirred at 0 °C until TLC (Alox, 5% MeOH / CH2Cl2) indicated full conversion. The THF was removed under reduced pressure and the residual aqueous solution was adjusted to pH = 6 using 4 N HCl. The solvent was removed under reduced pressure affording the product in quantitative yield, used in the next step without further purification.

6-((bis(pyridin-2-ylmethyl)amino)methyl)-*N*-methyl-*N*-((2S,3R,4R,5R)-2,3,4,5,6-pentahydroxyhexyl)nicotinamide (**ZN148**). 6-((bis(pyridin-2-ylmethyl)amino)methyl)nicotinic acid (**ZN146**) (415 mg, 1.24 mmol, 1.0 eq.) was dissolved in 10 mL dry DMF at room temperature. *N*-Methyl-*D*-Glucamine (363 mg, 1.86 mmol, 1.5 eq.), *N*-ethylcarbodiimide hydrochloride (EDCl) (356 mg, 1.86 mmol, 1.5 eq.), 1-Hydroxy-7-azabenzotriazol (HOAt) (253 mg, 1.86 mmol, 1.5 eq.) and *N*-methylmorpholine (NMM) (205 µL, 1.86 mmol, 1.5 eq.) were then added. The mixture was heated to 50 °C, kept at 16 h with stirring and then concentrated under reduced pressure. Purification of the resulting yellow-brown oil was achieved by way of dry column vacuum chromatography on C18 material, using a stepwise elution from 10% to 70% methanol in water affording 510.4 mg (81%) of product as an orange foam. The product appears as a syn/anti mixture regarding the amide bond. ^{1}H NMR (600 MHz, MeOD) δ 8.58 (s, 1H), 8.54 (s, 1H), 8.44 (d, *J* = 4.6 Hz, 4H), 7.93 (dd, *J* = 8.0, 1.7 Hz, 1H), 7.86 (dd, *J* = 8.0, 1.6 Hz, 1H), 7.80 (dd, *J* = 13.5, 6.3 Hz, 4H), 7.72 (dd, *J* = 15.9, 8.1 Hz, 2H), 7.68 (d, *J* = 7.9 Hz, 4H), 7.34 – 7.21 (m, *J* = 7.2, 3.8 Hz, 4H), 4.15 (dt, *J* = 8.1, 4.0 Hz, 1H), 4.07 – 3.96 (m, 1H), 3.95 – 3.83 (m, 12H), 3.83 – 3.46 (m, 13H), 3.14 (s, 3H), 3.08 (s, 3H), see fig. S5. ^{13}C NMR (151 MHz, MeOD) δ 172.0, 171.4, 161.8, 161.2, 160.0, 149.6, 148.5, 148.0, 138.8, 138.7, 137.7, 137.0, 132.7, 132.4, 125.0, 124.3, 124.1, 123.9, 115.3, 74.0, 73.4, 73.0, 72.9, 72.4, 71.7, 71.5, 71.1, 64.8, 64.7, 61.3, 61.2, 61.0, 60.9, 55.2, 52.5, 40.0, 33.8, see fig. S6. APCI-HRMS *e/z* calc. for $C_{26}H_{34}N_5O_6$: 512.2504, found 512.2502 [M+H].

Methyl 6-((dibenzylamino)methyl)nicotinate (**OAHA7137**). Dibenzylamine (986 mg, 5.0 mmol, 1.0 eq) was dissolved in THF (100 mL) at room temperature and mixed with methyl 6-(bromomethyl)nicotinate (1150 mg, 5.0 mmol, 1.0 eq) and DIPEA (1.275 mL, 7.5 mmol, 1.5 eq). The mixture was stirred at room temperature for 24 hours before it was filtered through a short plug of celite, concentrated under reduced pressure, re-dissolved in Et_2O (100 mL) and filtered again through celite. The filtrate was collected and concentrated under reduced pressure to give 1660 mg (96%) of the titled product, which was used in the next step without further purification. ^{1}H NMR (400 MHz, DMSO-d_6) δ 8.98 (d, *J* = 2.2 Hz, 1H), 8.27 (dd, *J* = 8.2, 2.3 Hz, 1H), 7.72 (d, *J* = 8.0 Hz, 1H), 7.39 (d, *J* = 7.1 Hz, 4H), 7.33 (t, *J* = 7.5 Hz, 4H), 7.24 (t, *J* = 7.2 Hz, 2H), 3.86 (s, 3H), 3.71 (s, 2H), 3.57 (s, 4H), see fig. S7. ^{13}C NMR (101 MHz, DMSO) δ 165.2, 164.3, 149.4, 138.6, 137.3, 128.6, 128.3, 127.0, 124.0, 122.3, 58.7, 57.4, 52.3, see fig. S8. APCI-HRMS e/z calc. for $C_{22}H_{23}N_2O_2$: 347.1754, found 347.1754 [M+H].

6-((dibenzylamino)methyl)-*N*-methyl-*N*-((2*S*,3*R*,4*R*,5*R*)-2,3,4,5,6-pentahydroxyhexyl)nicotinamide (**ZN222**). The methyl 6-((benzyl(pyridin-2-ylmethyl)amino)methyl)nicotinate (**OAHA7137**) (1730 mg, 5.0 mmol, 1.0 eq.) was dissolved in 10 mL THF and lithium hydroxide monohydrate (1049 mg, 25.0 mmol, 5.0 eq) was added with the aid of 2 mL H_2O at room temperature. The reaction was stirred for 16h before 1M HCl (20 mL, 20 mmol, 4.0 eq) was added drop wise. The neutralized reaction was then concentrated under

reduced pressure before it was re-dissolved in 10 mL dry DMF at room temperature. *N*-Methyl-*D*-Glucamine (976 mg, 5.0 mmol, 1.0 eq.), EDCl (957 mg, 5.0 mmol, 1.0 eq.), HOAt (681 mg, 5.0 mmol, 1.0 eq.) and NMM (1.1 mL, 10.0 mmol, 2.0 eq.) were then added. The mixture was heated to 50 °C, kept for 48 h with stirring and then concentrated under reduced pressure. Purification of the product was achieved by dry column vacuum chromatography on C18 bondesil material, using a stepwise elution from 10% to 80% methanol in water affording 0.457 g (0.9 mmol, 18%) of product. ^{1}H NMR (400 MHz, MeOD) δ 8.57 (s, 1H), 8.51 (s, 1H), 7.96 (d, *J* = 7.9 Hz, 1H), 7.87 (d, *J* = 7.3 Hz, 1H), 7.73 (d, *J* = 8.1 Hz, 2H), 7.40 (d, *J* = 7.4 Hz, 8H), 7.30 (t, *J* = 6.7 Hz, 8H), 7.22 (t, *J* = 7.0 Hz, 4H), 4.21 – 4.11 (m, 1H), 4.07 – 3.98 (m, 1H), 3.85 – 3.44 (m, 26H), 3.14 (s, *J* = 12.0 Hz, 3H), 3.07 (s, 3H), see fig. S9. ^{13}C NMR (101 MHz, MeOD) δ 172.1, 171.5, 162.9, 162.4, 148.2, 147.7, 140.2, 137.8, 137.1, 132.5, 132.2, 130.0, 129.4, 128.2, 124.0, 123.8, 74.0, 73.5, 73.0, 72.9, 72.4, 71.6, 71.5, 71.0, 64.8, 60.2, 60.0, 59.6, 59.4, 55.2, 52.4, 40.0, 33.8, see fig. S10. APCI-HRMS *e/z* calc. for $C_{28}H_{36}N_{3}O_{6}$: 510.2599, found 510.2599 [M+H].

N-benzyl-1-(pyridin-2-yl)methanamine (**OAHA7139**). Benzylamine (1000 mg, 9.33 mmol, 1.0 eq.) was dissolved in absolute ethanol (50 mL) at room temperature and mixed with 2-pyridine carboxaldehyde (1000 mg, 9.34 mmol, 1.0 eq.). The red solution was heated to 50 °C and stirred for 24 hours before solid $NaBH_4$ (2500 mg, 66.09 mmol, 7.08 eq) was added in one portion and the slurry was stirred for 4 days at 50 °C. The crude mixture was then concentrated under reduced pressure to give a pale-yellow solid which was dissolved in 1M K_2CO_3 solution (100 mL) and extracted with dichloromethane (3x50 mL). The combined organic fractions were pooled, dried over Na_2SO_4, filtered and concentrated under reduced pressure to give 1826 mg (99%) of the titled product as a yellow oil which needed no further purification. ^{1}H NMR (400 MHz, DMSO-d6) δ 8.70 – 8.21 (m, 1H), 7.75 (td, J = 7.7, 1.8 Hz, 1H), 7.46 (d, J = 7.8 Hz, 1H), 7.39 – 7.28 (m, 5H), 7.27 – 7.19 (m, 2H), 3.78 (s, 2H), 3.72 (s, 2H), 2.70 (s, 1H), see fig. S11. ^{13}C NMR (101 MHz, DMSO) δ 160.3, 148.7, 140.7, 136.4, 128.1, 127.9, 126.5, 121.8, 121.7, 53.8, 52.4, see fig. S12. ECI-HRMS e/z calc. for $C_{13}H_{15}N_{2}$: 199.1230, found 199.1230 [M+H].

Methyl 6-((benzyl(pyridin-2-ylmethyl)amino)methyl)nicotinate (**OAHA7145**). *N*-benzyl-1-(pyridin-2-yl)methanamine (**OAHA7139**) (991 mg, 5.0 mmol, 1.0 eq) was dissolved in THF (100 mL) at room temperature and mixed with methyl 6-(bromomethyl)nicotinate (1150 mg, 5.0 mmol, 1.0 eq) and DIPEA (1.36 mL, 8.0 mmol, 1.6 eq). The mixture was stirred at room temperature for 24 hours before it was filtered through a short plug of celite, concentrated under reduced pressure, re-dissolved in Et_2O (100 mL) and filtered again through celite. The filtrate was collected and concentrated under reduced pressure to give 1660 mg (96%) of the titled product which was used in the next step without further purification. APCI-HRMS e/z calc. for $C_{21}H_{22}N_{3}O_{2}$: 348.1707, found 348.1707 [M+H].

6-((benzyl(pyridin-2-ylmethyl)amino)methyl)-*N*-methyl-N-((2S,3R,4R,5R)-2,3,4,5,6-pentahydroxyhexyl)nicotinamide (**ZN223**). Methyl 6-((benzyl(pyridin-2-ylmethyl)amino)methyl)nicotinate (**OAHA7145**) (973 mg, 2.80 mmol, 1.0 eq.) was dissolved in 10 mL THF and lithium hydroxide monohydrate (588 mg, 14.0 mmol, 5.0 eq) was added with the aid of 2 mL H_2O at room temperature. The reaction was stirred for 16h before 1M HCl (11.2 mL, 11.2 mmol, 4.0 eq) was added drop wise. The neutralized reaction was then concentrated under reduced pressure before it was re-dissolved in 10 mL dry DMF at room temperature. *N*-Methyl-*D*-Glucamine (547 mg, 2.80 mmol, 1.0 eq.), EDCl (537 mg, 2.80 mmol, 1.0 eq.), HOAt (381 mg, 2.80 mmol, 1.0 eq.) and NMM (616 µL, 5.60 mmol, 2.0 eq.) were then added. The mixture was heated to 50 °C, kept for 48h with stirring and then concentrated under reduced pressure. Purification of the product was achieved by way of dry column vacuum chromatography on C18

bondesil material, using a stepwise elution from 10% to 70% methanol in water affording 0.72 g (1.4 mmol, 50%) of product. ^{1}H NMR (400 MHz, MeOD) δ 8.58 (s, 1H), 8.53 (d, *J* = 1.1 Hz, 1H), 8.43 (dd, *J* = 4.9, 0.7 Hz, 2H), 7.95 (dd, *J* = 8.0, 1.8 Hz, 1H), 7.87 (dd, *J* = 8.0, 1.7 Hz, 1H), 7.80 (t, *J* = 7.6 Hz, 2H), 7.72 (dd, *J* = 7.7, 5.8 Hz, 2H), 7.68 (d, *J* = 7.9 Hz, 2H), 7.40 (d, *J* = 7.4 Hz, 4H), 7.35 – 7.25 (m, 6H), 7.25 – 7.17 (m, 2H), 4.16 (dt, *J* = 8.1, 4.0 Hz, 1H), 4.03 (dd, *J* = 6.1, 3.8 Hz, 1H), 3.88 – 3.76 (m, 10H), 3.76 – 3.43 (m, 16H), 3.14 (s, *J* = 6.4 Hz, 3H), 3.07 (s, 3H), see fig S13. ^{13}C NMR (101 MHz, MeOD) δ 172.0, 171.4, 162.3, 161.7, 160.4, 149.4, 148.4, 147.8, 139.7, 138.7, 137.8, 137.1, 132.6, 132.3, 130.1, 129.4, 128.4, 124.8, 124.2, 123.8, 73.9, 73.5, 73.0, 72.9, 72.4, 71.6, 71.5, 71.0, 64.7, 60.9, 60.7, 60.6, 60.4, 60.0, 59.8, 55.2, 52.4, 40.0, 33.8, see fig. S14. APCI-HRMS *e/z* calc. for $C_{27}H_{35}N_4O_6$: 511.2551, found 511.2551 [M+H].

Methyl 4-((bis(pyridin-2-ylmethyl)amino)methyl)benzoate (**OAHA7159**). Dipicolylamine (1.84 mL, 10.0 mmol, 1.0 eq) was dissolved in THF (150 mL) at room temperature and mixed with methyl bromomethylbenzoate (2.29 g, 10.0 mmol, 1.0 eq) and DIPEA (2.7 mL, 16 mmol, 1.6 eq). The mixture was stirred at room temperature for 24 hours before it was filtered through a short plug of celite, concentrated under reduced pressure, re-dissolved in Et_2O (200 mL) and filtered again through celite. The filtrate was collected and concentrated under reduced pressure to give 3.68 g of the titled product which was used in the next step without further purification. ^{1}H NMR (400 MHz, DMSO-d_6) δ 8.49 (ddd, *J* = 4.8, 1.8, 0.9 Hz, 1H), 7.97 – 7.87 (m, 1H), 7.77 (td, *J* = 7.6, 1.8 Hz, 1H), 7.56 (dt, *J* = 7.9, 1.3 Hz, 2H), 7.25 (ddd, *J* = 7.5, 4.8, 1.2 Hz, 1H), 3.83 (s, 1H), 3.72 (d, *J* = 2.0 Hz, 3H), see fig. S15. ^{13}C NMR (101 MHz, DMSO) δ 166.1, 158.8, 148.8, 144.7, 136.6, 129.1, 128.8, 128.3, 122.6, 122.2, 59.2, 57.1, 52.0, see fig. S16. APCI-HRMS e/z calc. for $C_{21}H_{22}N_3O_2$: 348.1707, found 348.1707 [M+H].

4-((bis(pyridin-2-ylmethyl)amino)methyl)-*N*-methyl-*N*-((2S,3R,4R,5R)-2,3,4,5,6-pentahydroxyhexyl)benzamide (**ZN228**). Methyl 4-((bis(pyridin-2-ylmethyl)amino)methyl)benzoate (0.560 g, 1.61 mmol, 1.0 eq.), (**OAHA7159**), was dissolved in a mixture of 10 mL THF and 10 mL H_2O at room temperature. To this solution was added LiOH.H_2O (0.203 g, 4.84 mmol, 3 eq.) and the reaction progress monitored by TLC on alumina using 5% MeOH in CH_2Cl_2. After 16 h, the crude reaction mixture was concentrated under reduced pressure and the residue dissolved in 5 mL dest. H_2O. The pH of the basic solution was adjusted to 4 with 2M HCl and the mixture concentrated under reduced pressure. The obtained 4-((bis(pyridin-2-ylmethyl)amino)methyl)benzoic acid (0.537 g, 1.61 mmol, 1.0 eq.) was dissolved in 20 mL dry DMF at room temperature. *N*-Methyl-*D*-Glucamine (0.471 g, 2.41 mmol, 1.5 eq.), EDCl (0.462 g, 2.41 mmol, 1.5 eq.), HOAt (0.328 g, 2.41 mmol, 1.5 eq.) and NMM (0.266 mL, 2.41 mmol, 1.5 eq.) were then added. The mixture was heated to 50 °C, kept for 16 h with stirring and then concentrated under reduced pressure. Purification of the product was achieved by dry column vacuum chromatography on C18 bondesil material, using a stepwise elution from 10% to 90% methanol in water affording 0.108 g (0.212 mmol, 13%) of product. ^{1}H NMR (400 MHz, MeOD) δ 8.43 (d, J = 4.5 Hz, 4H), 7.81 (t, J = 7.3 Hz, 4H), 7.68 (d, J = 7.8 Hz, 4H), 7.46 (ddd, J = 29.4, 16.9, 8.5 Hz, 8H), 7.33 – 7.22 (m, 4H), 4.19 – 4.10 (m, 1H), 3.98 (dd, J = 4.4, 3.7 Hz, 1H), 3.79 (s, J = 16.3 Hz, 10H), 3.75 – 3.42 (m, 15H), 3.13, 3.05 (2xs, 6H), see fig. S17. ^{13}C NMR (101 MHz, MeOD) δ 174.9, 174.3, 160.4, 149.5, 142.1, 141.5, 138.7, 136.6, 136.4, 130.1, 129.9, 128.6, 128.1, 124.8, 123.9, 74.1, 73.6, 73.0, 72.9, 72.6, 71.7, 71.6, 71.4, 64.8, 64.7, 60.9, 60.7, 59.5, 59.4, 55.4, 52.3, 40.1, 34.0, see fig. S18. APCI-HRMS e/z calc. for $C_{27}H_{35}N_4O_6$: 511.2551, found 511.2551 [M+H].

References:

1. Humphreys KJ, Karlin KD, Rokita SE. Efficient and specific strand scission of DNA by a dinuclear copper complex: comparative reactivity of complexes with linked tris(2-pyridylmethyl)amine moieties. J Am Chem Soc. 2002;124(21):6009-19.

SUPPLEMENTAL FIGURES

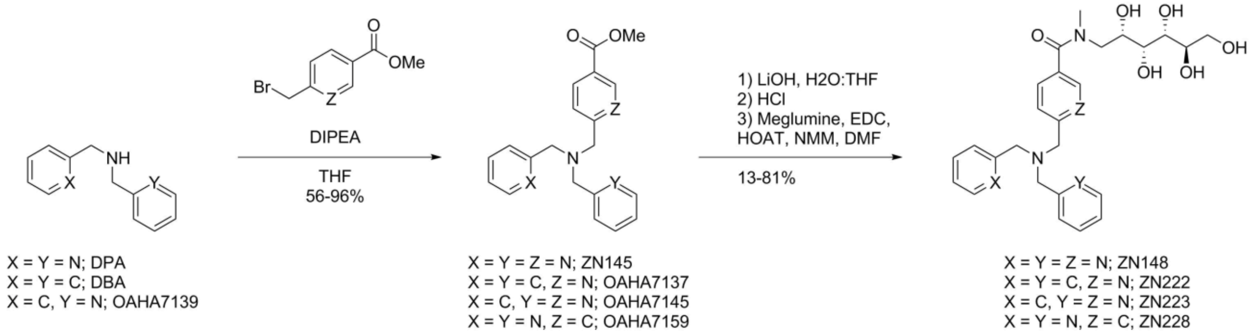

Fig. S1 Synthetic pathway towards ZN148, ZN222, ZN223 and ZN228. DPA = N,N'-dipicolylamine. DBA = N,N'-dibenzylamine. DIPEA = Diisopropylethylamine. THF = Tetrahydrofuran. EDC = 1-Ethyl-3-(3-dimethylaminopropyl)carbodiimide, HOAT = 1-Hydroxy-7-azabenzotriazole, NMM = N-methylmorpholine. DMF = Dimethylformamide.

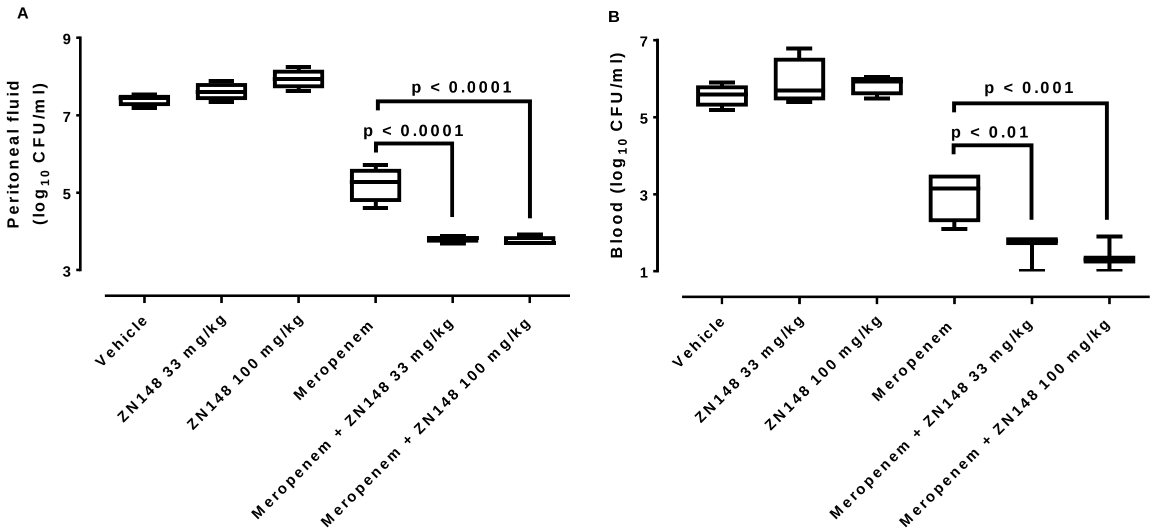

Fig. S2 *In vivo* efficacy of meropenem and meropenem-ZN148 combinations. Neutropenic NMRI mice were inoculated intraperitoneally with ~5x10^6 CFU of NDM-1-producing *K. pneumoniae* 50752501 (meropenem MIC = 64 mg/L). Mice were treated by subcutaneously injection in the neck region with vehicle (PBS), ZN148 (33 or 100 mg/kg), meropenem (33 mg/kg) or meropenem (33 mg/kg) + ZN148 (33 or 100 mg/kg). Vehicle and ZN148 were given one-hour post-inoculation while meropenem were given 1.5 hrs post-inoculation. Colony counts in peritoneal fluid (A) and blood (B) were determined five hrs post-inoculation. Four mice were included in each group. Groups were analyzed with ANOVA Dunnett´s multiple comparisons test in GraphPad Prism 7.04 (GraphPad Software Inc, USA). *P*-values <0.05 were considered statistically significant.

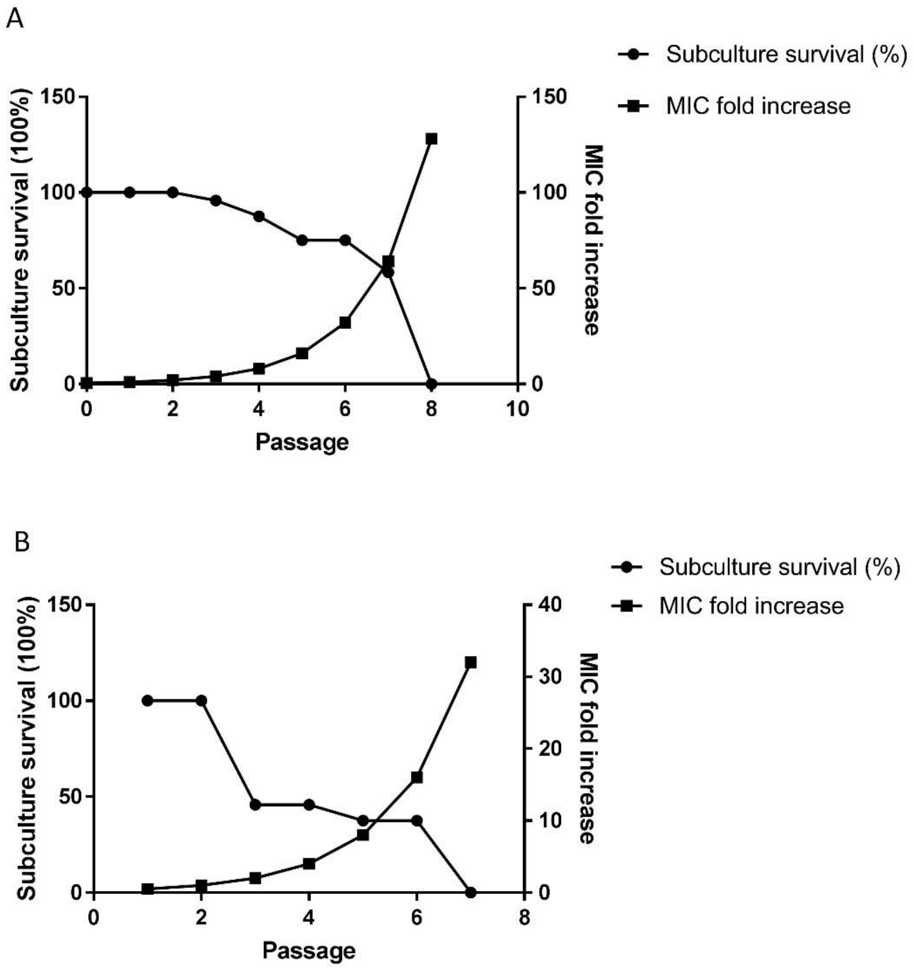

Fig. S3. Serial passaging of NDM-1-producing *K. pneumoniae* K66-45 on increasing concentrations of meropenem. Passaging was performed in Mueller Hinton Broth II medium in the presence of (A) 50 µM and (B) 100 µM ZN148. Evolved cultures reached a maximum of 64x original MIC (8 mg/L) (A) and 16x original MIC (2 mg/L) (B), respectively, before growth was no longer observed (8 passages (A) and 6 passages (B), respectively).

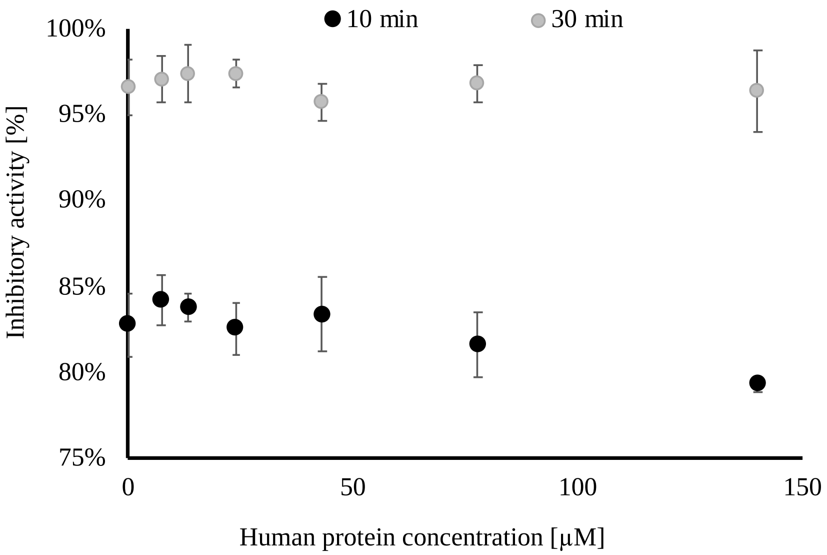

Fig. S4 Binding of ZN148 to human serum albumin and α_1-acid glycoprotein. 500 µM ZN148 in 50 mM HEPES buffer, pH 7.5 was incubated with human serum albumin and α_1-acid glycoprotein (ratio 24:1) for 12 min. Inhibitory activity of unbound ZN148 was subsequently measured by incubation for 10 and 30 min with VIM-2 and adding nitrocefin as a reporter substrate. Data is presented as percent inhibitory activity of ZN148 compared to control (VIM-2 alone). Error bars represent standard deviation.

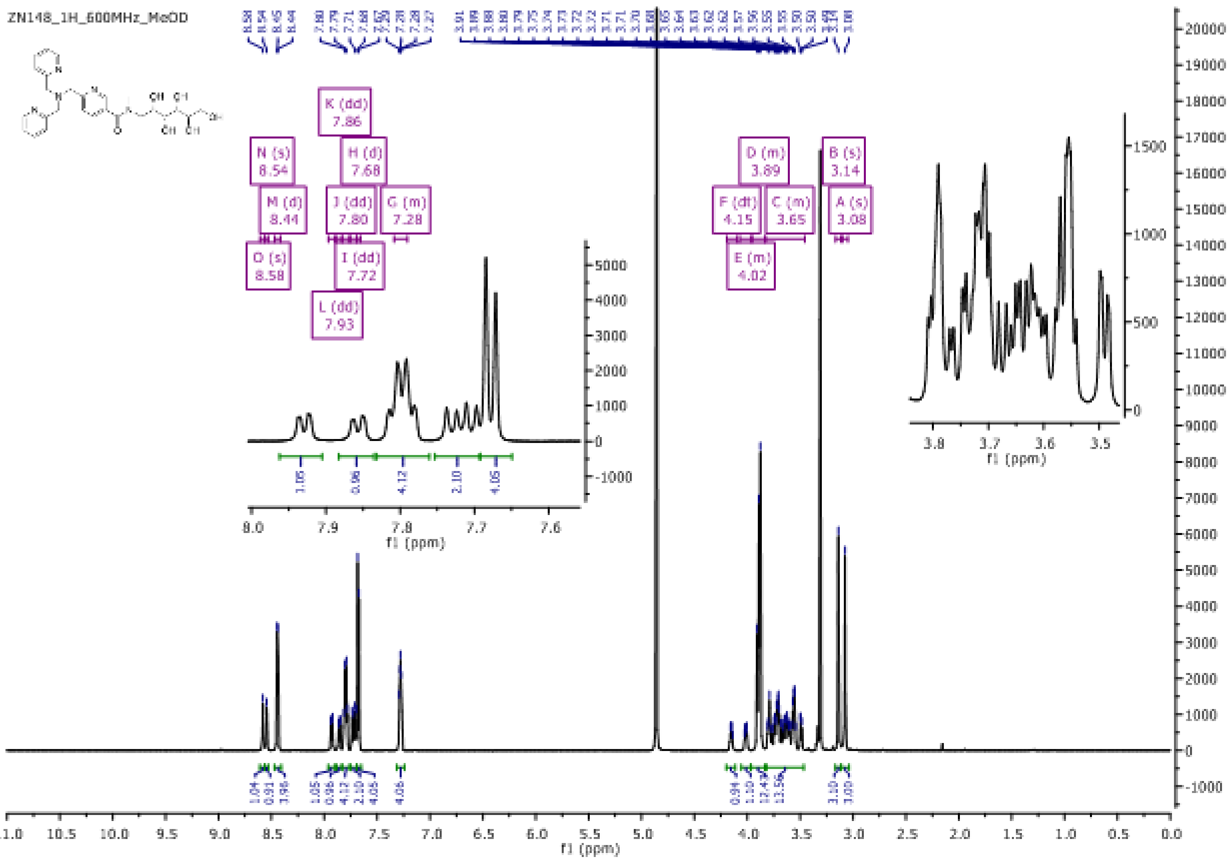

Fig. S5 ^{1}H NMR of ZN148 in D_4-MeOH.

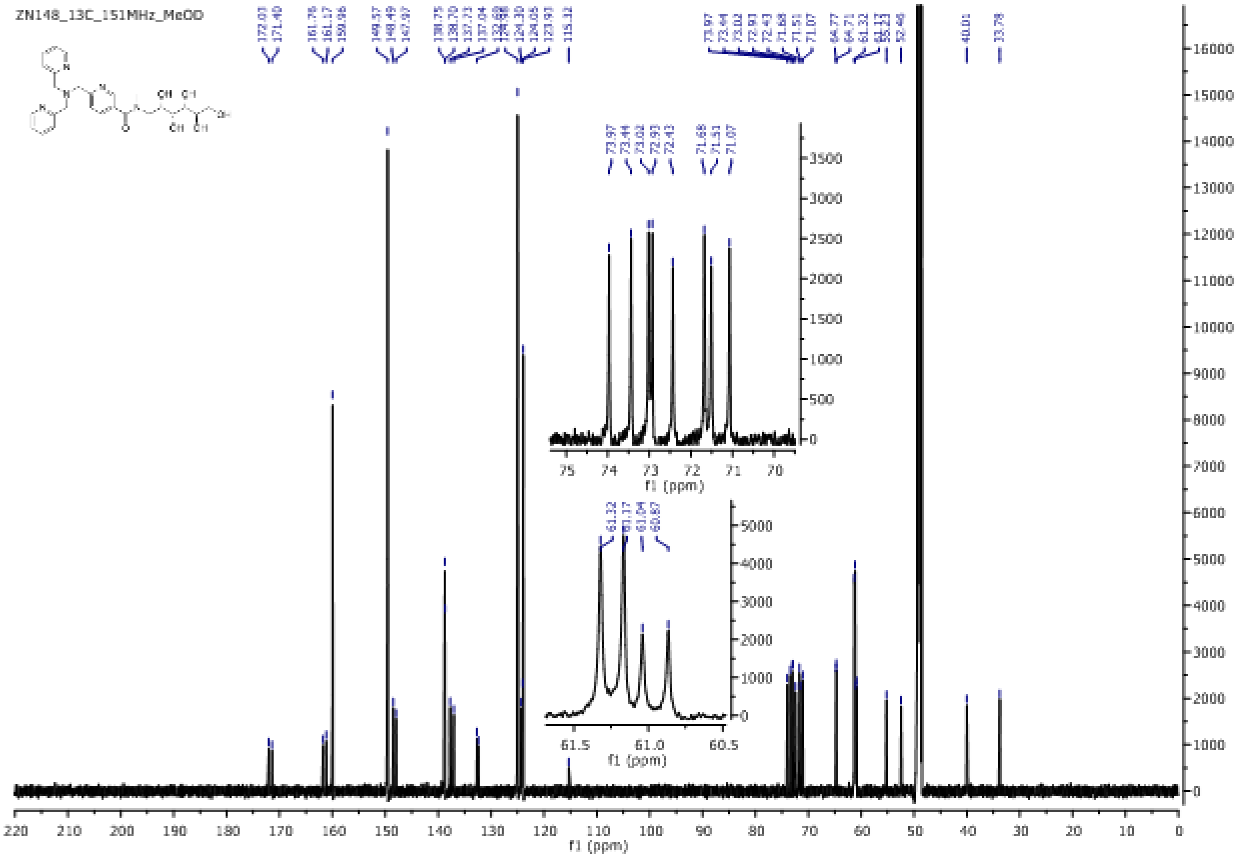

Fig. S6 ^{13}C NMR of ZN148 in D_4-MeOH.

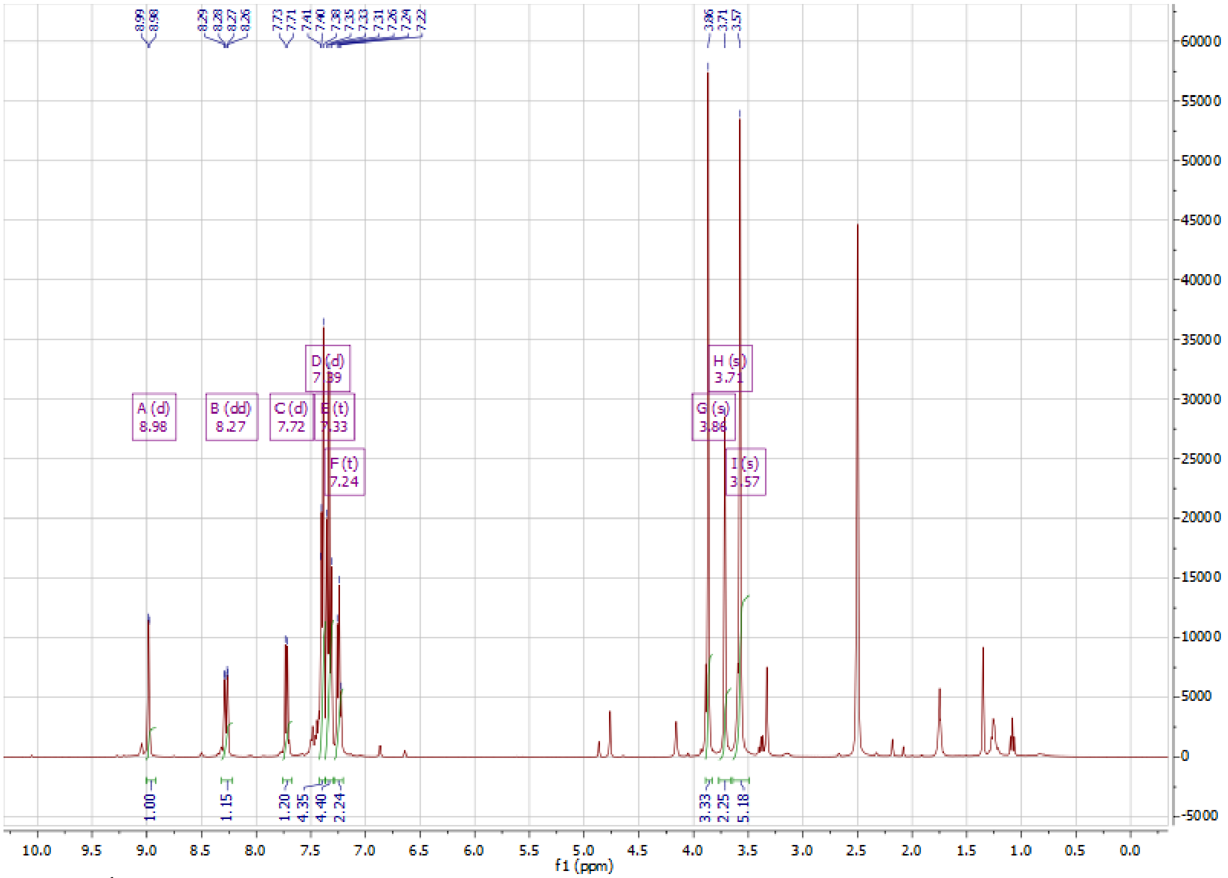

Fig. S7 ^{1}H NMR of OAHA7137 in D_6-DMSO.

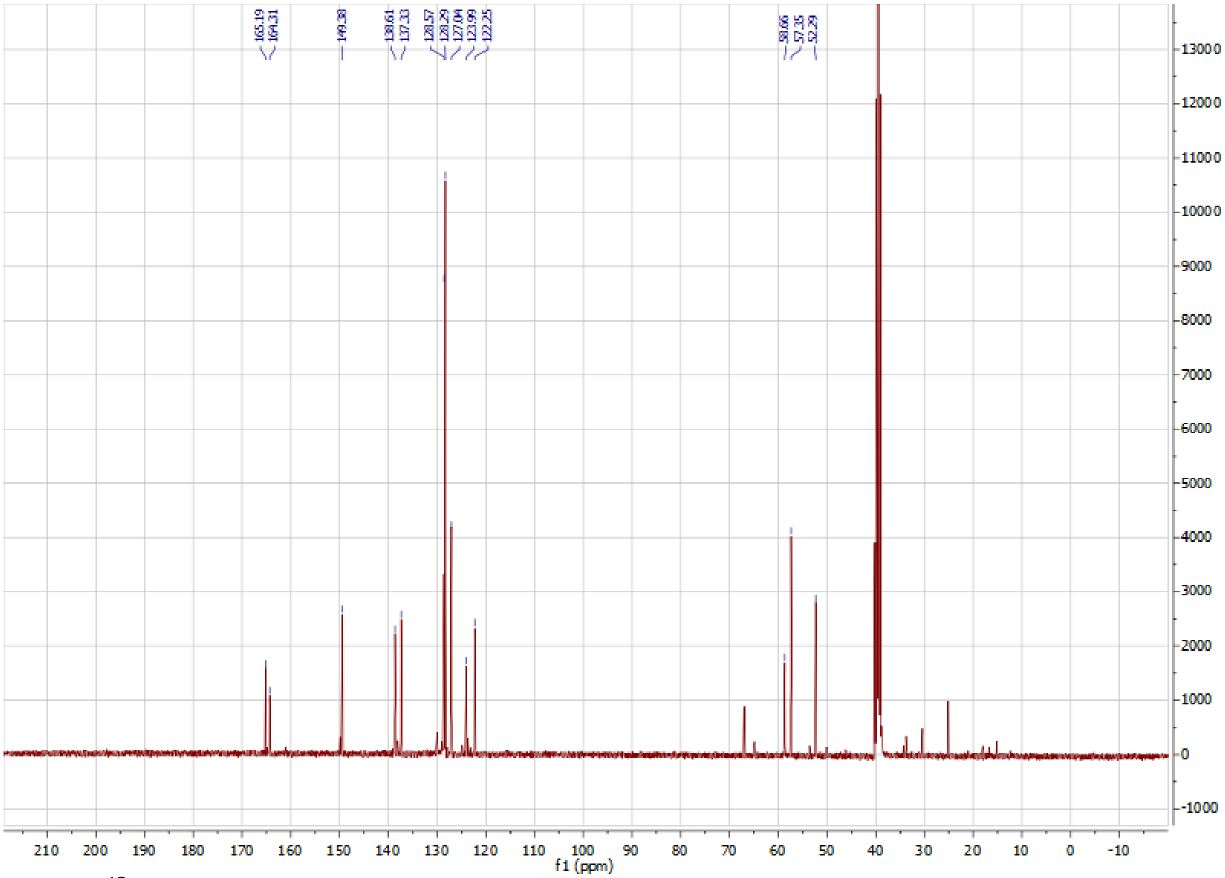

Fig. S8 ^{13}C NMR of OAHA7137 in D_6-DMSO.

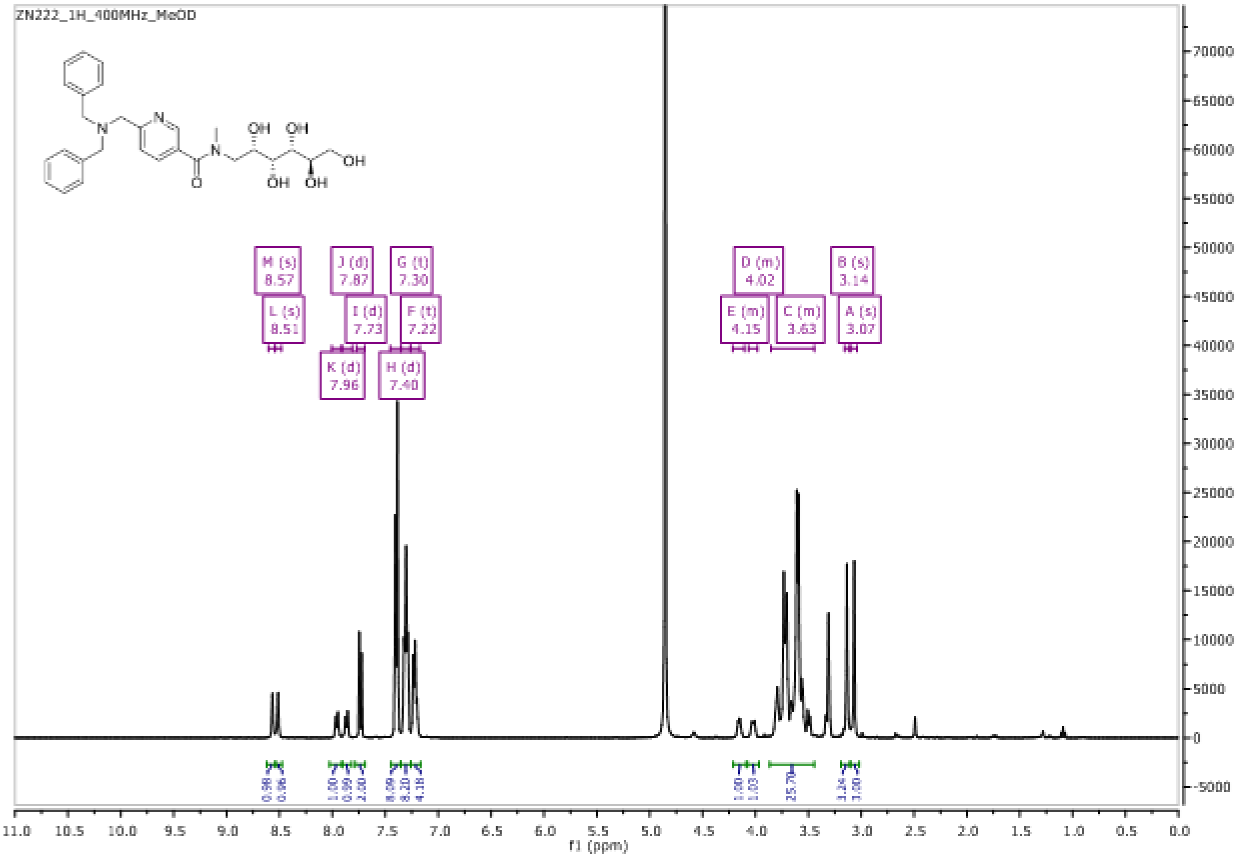

Fig. S9 ^{1}H NMR of ZN222 in D_4-MeOH.

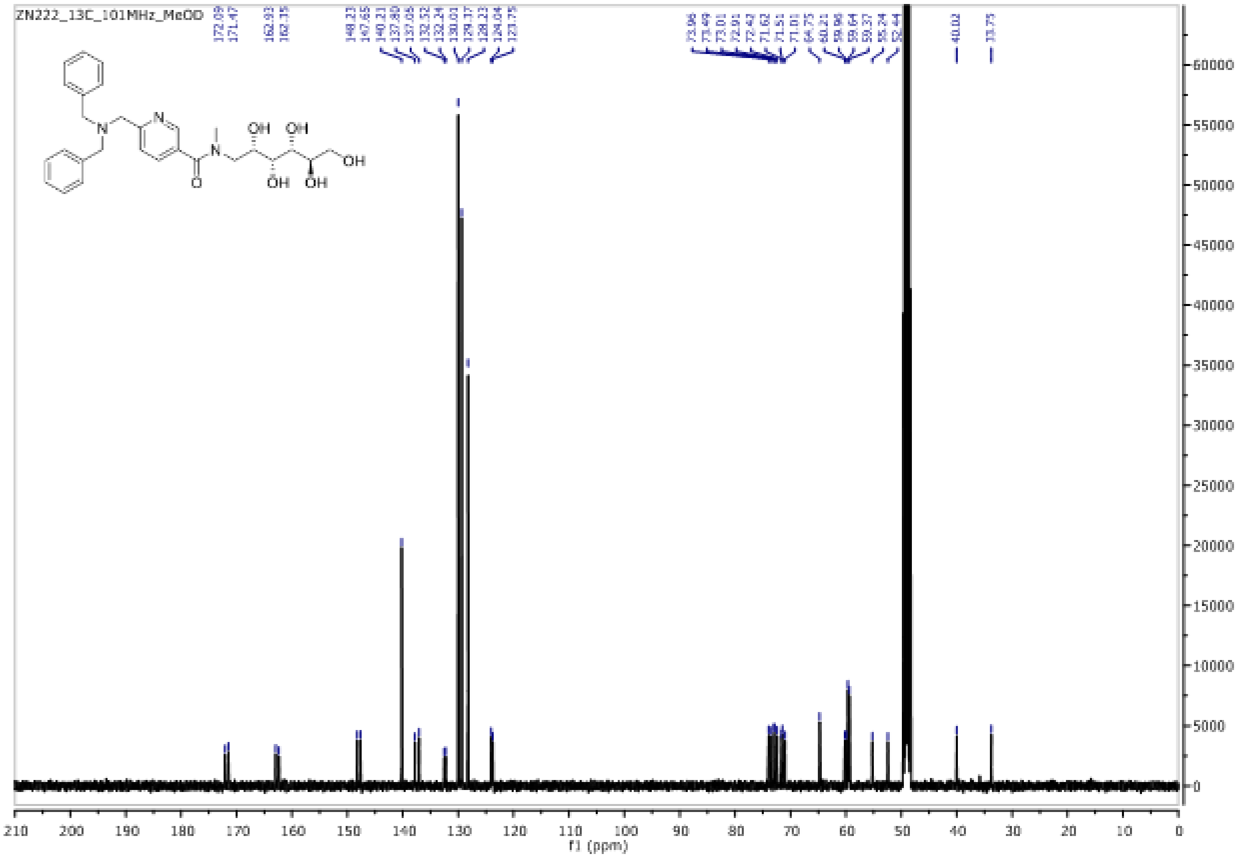

Fig. S10 ^{13}C NMR of ZN222 in D_4-MeOH.

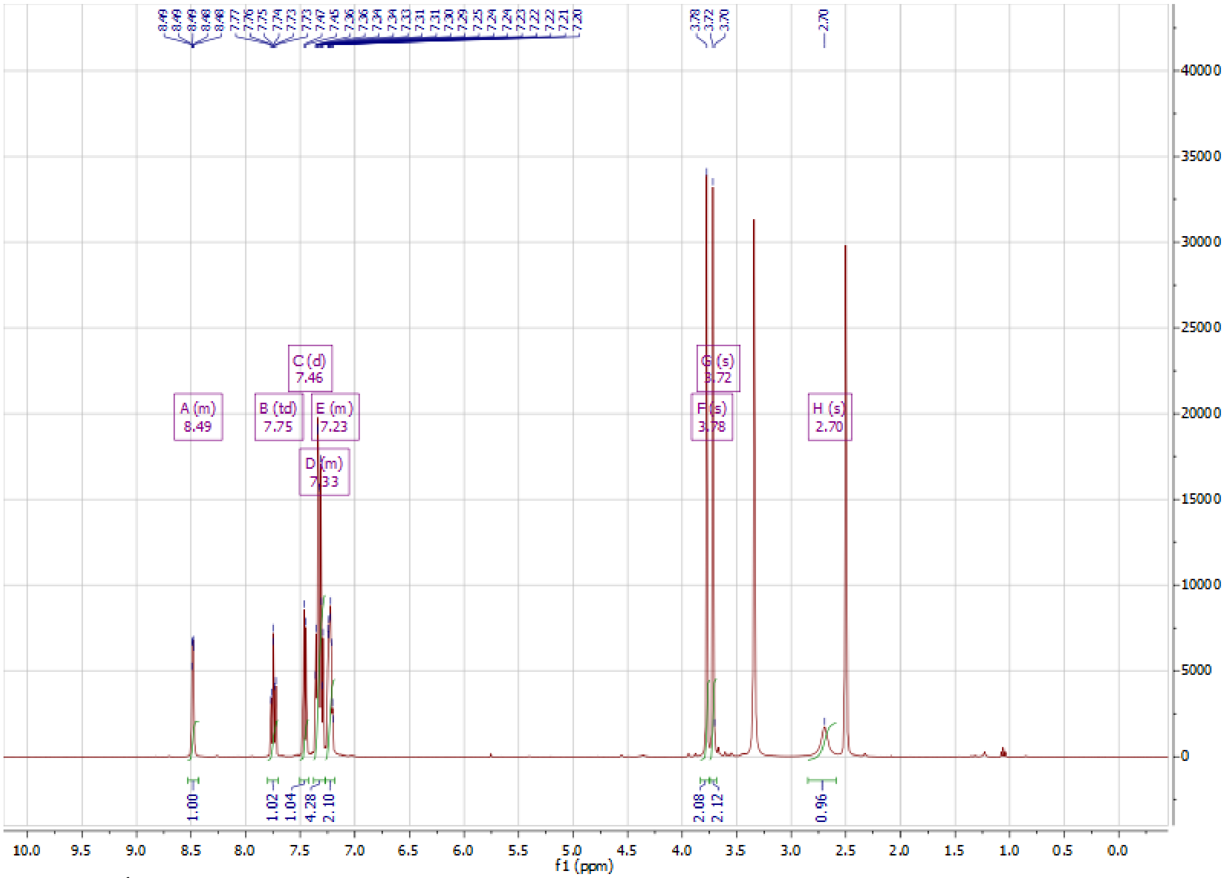

Fig. S11 ^{1}H NMR of OAHA7139 in D_6-DMSO.

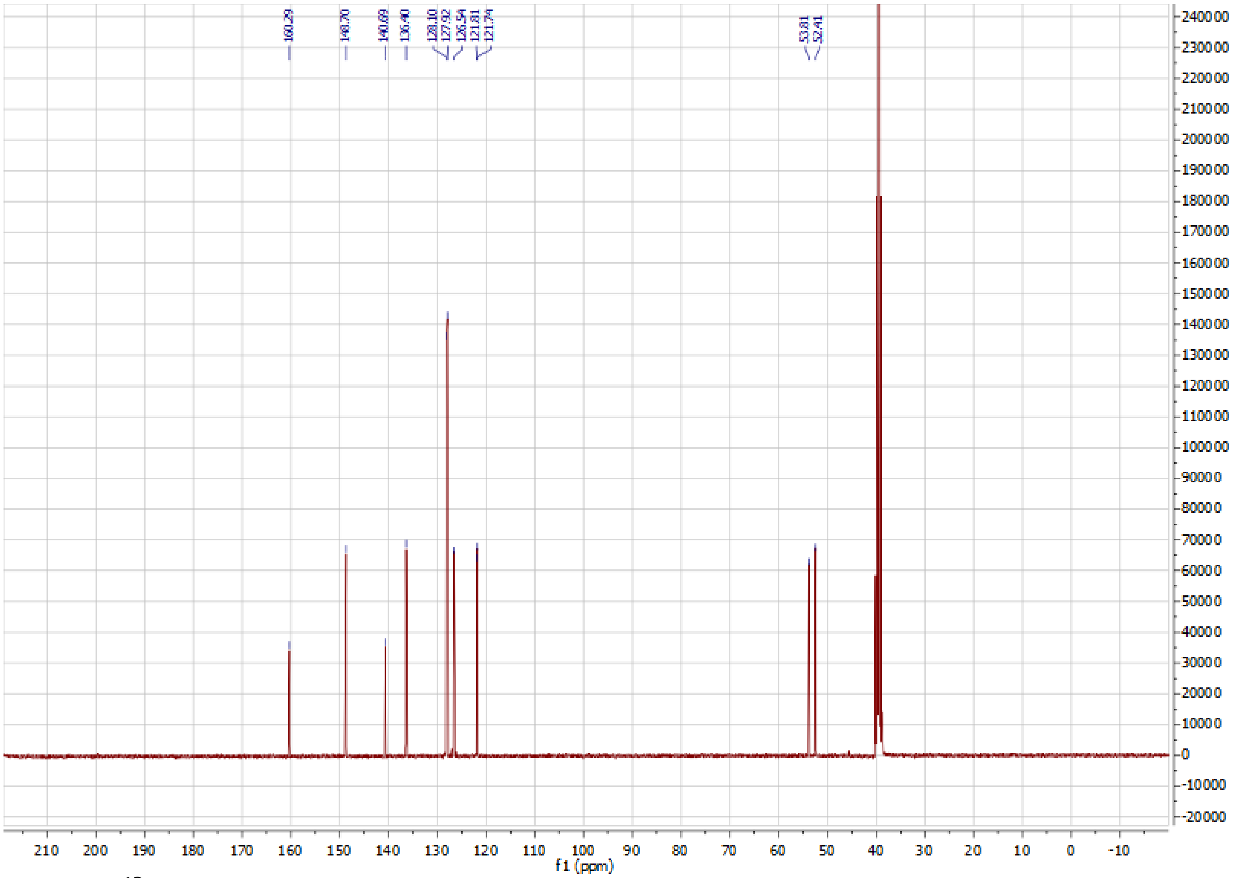

Fig. S12 ^{13}C NMR OF OAHA7139 in D_6-DMSO.

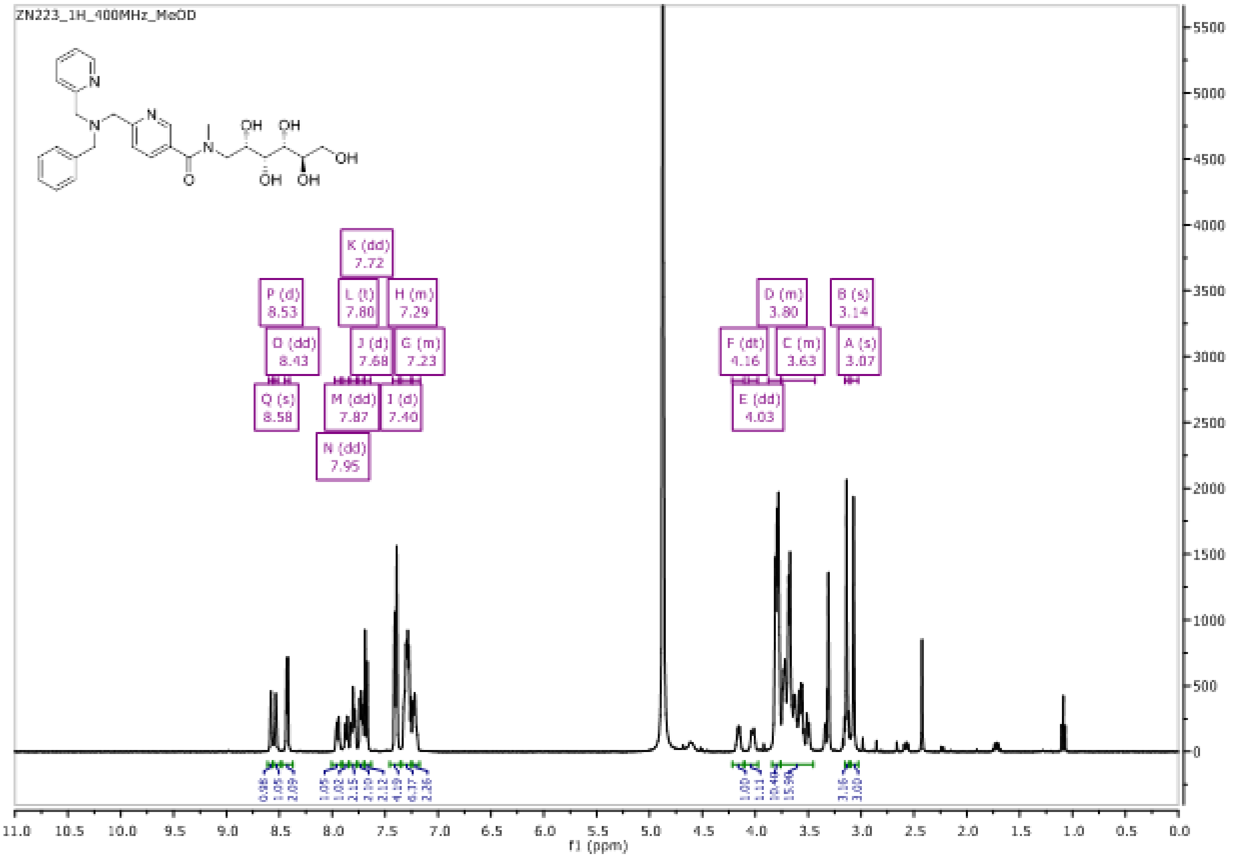

Fig. S13 ^{1}H NMR OF ZN223 in D_4-MeOH.-1HNMR.

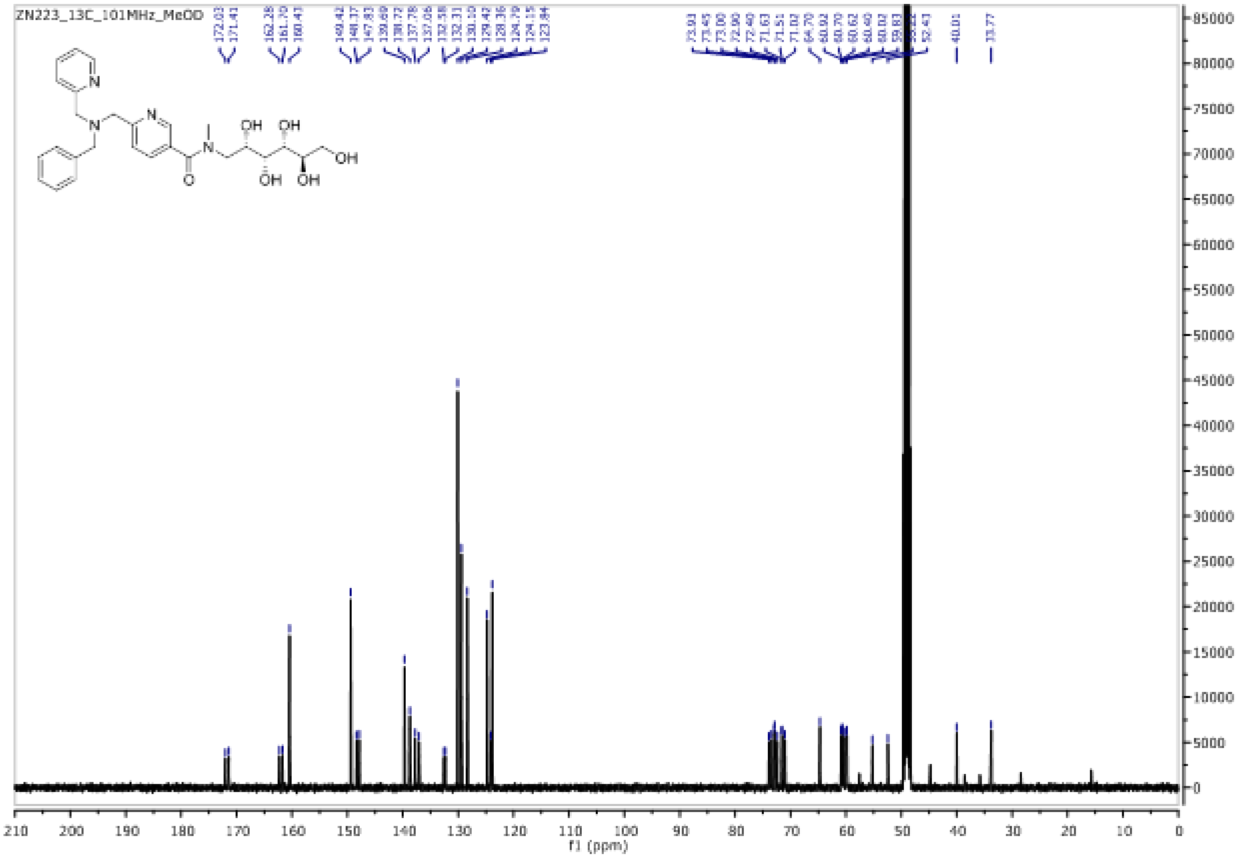

Fig. S14 ^{13}C **NMR OF ZN223 in** D_4**-MeOH.**

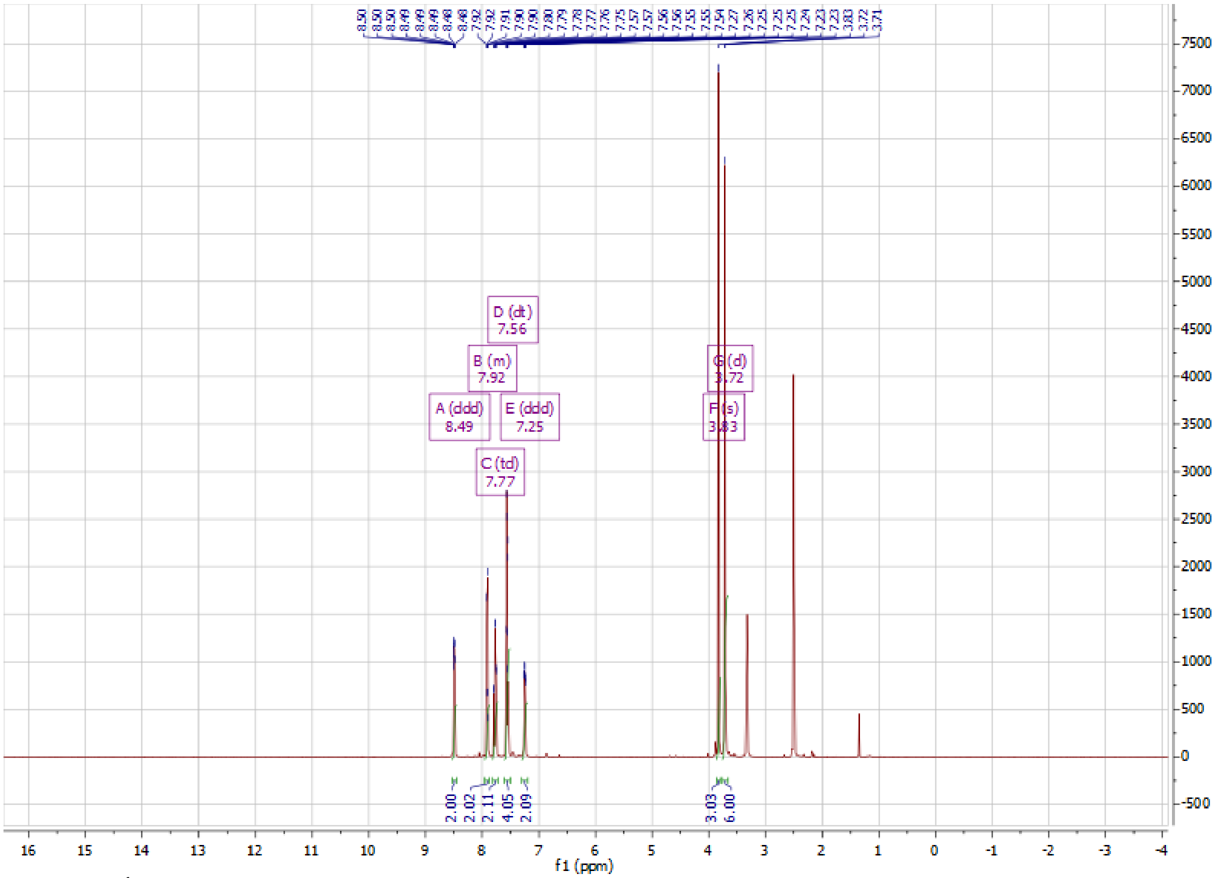

Fig. S15 ^{1}H NMR of OAHA7159 in D_6-DMSO.

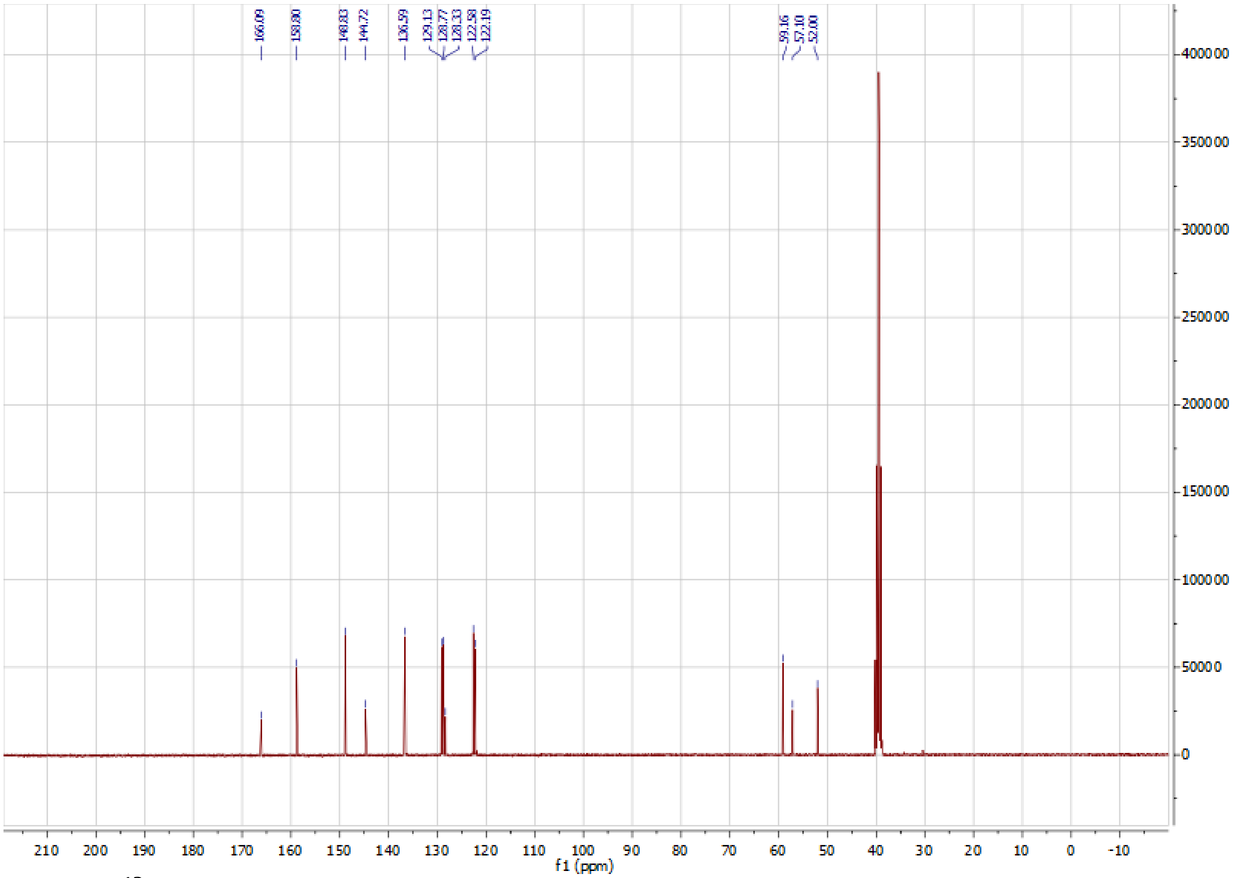

Fig. S16 ^{13}C NMR of OAHA7159 in D_6-DMSO.

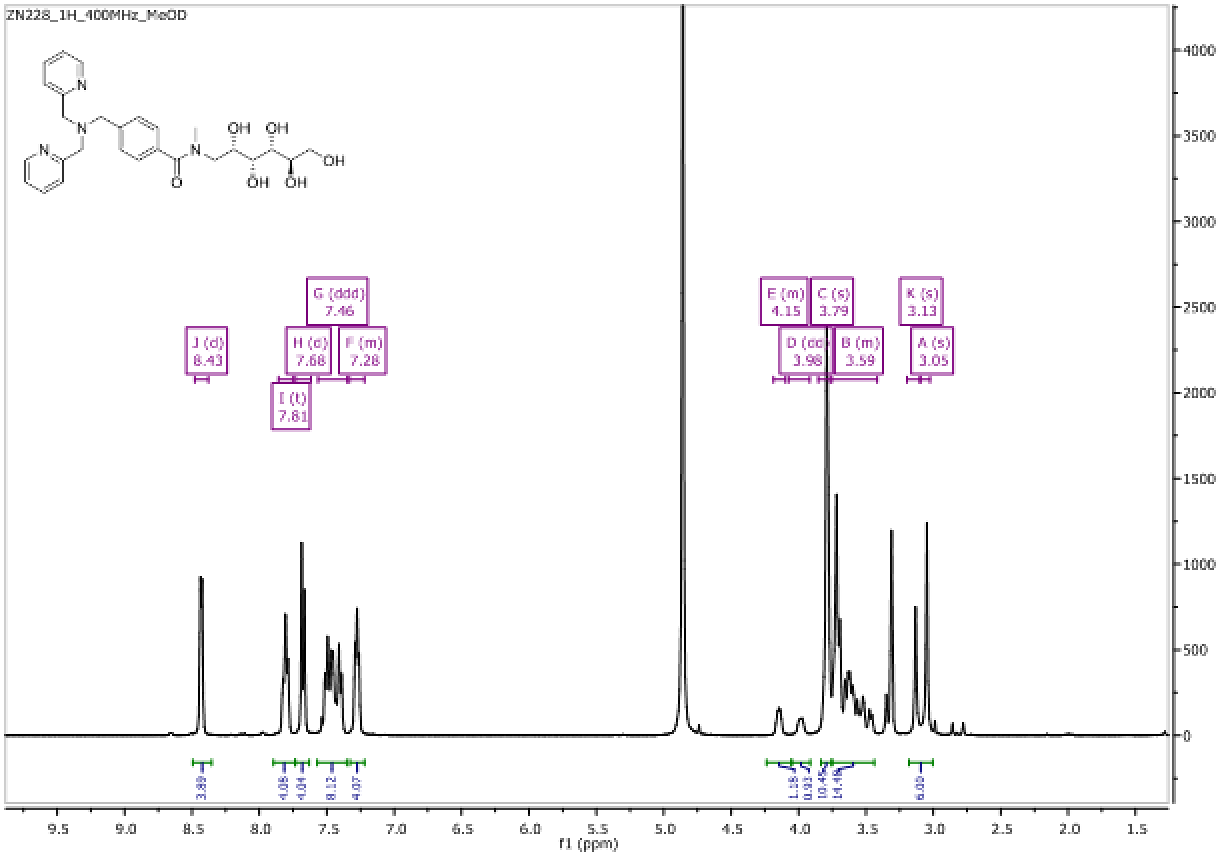

Fig. S17 ^{1}H NMR of ZN228 in D_4-MeOH.

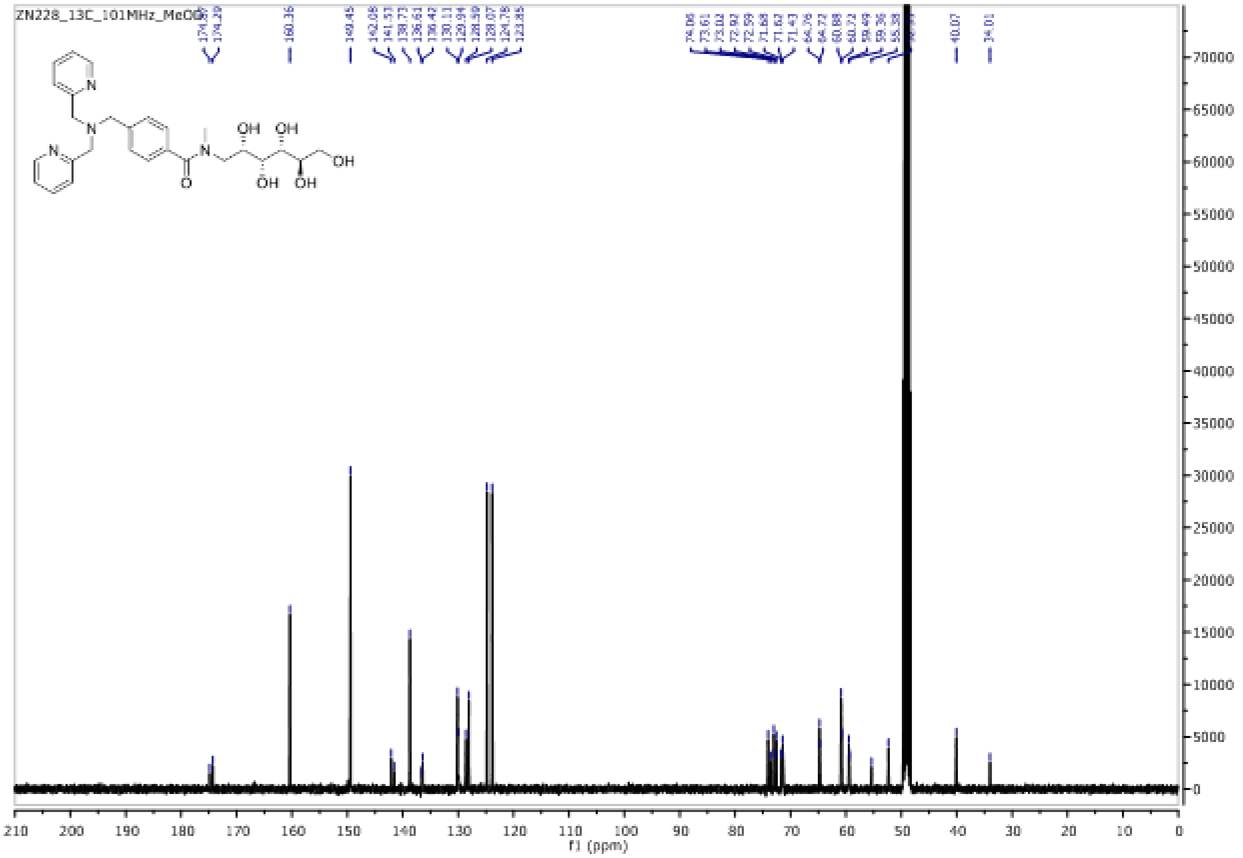

Fig. S18 ^{13}C NMR of ZN228 in D_4-MeOH.

SUPPLEMENTAL TABLES

Table S1.
MIC of meropenem alone and meropenem +/- 50 µM TPA or ZN148, and IC_{50} values of TPA and ZN148 against HepG2 cells.

	Meropenem MIC (mg/L)		IC_{50} (µM)	
Compound	*K. pneumoniae* K66-45 (NDM-1)	*P. aeruginosa* K34-7 (VIM-2)	Compound	HepG2
Meropenem alone	32-64	32-64		
Meropenem-TPA	0.125	1	TPA	9.75±0.86
Meropenem-ZN148	0.125	1	ZN148	>100

Table S2.

Meropenem (MEM) MIC distributions +/- 50 µM ZN148 against MBL-producing *Enterobacterales* (*n*=234).

		No. of strains with indicated MIC (mg/L)											
		≤0.03	0.06	0.125	0.25	0.5	1	2	4	8	16	32	≥64
E. coli with NDM (*n*=102)[a]	MEM alone						2		6	2	8	19	65
	MEM-ZN148	32	33	23	11	1	1			1			
E. coli with VIM (*n*=7)	MEM alone					1				3	2	1	
	MEM-ZN148	3	1	3									
E. coli with IMP (*n*=3)	MEM alone								1		2		
	MEM-ZN148	2		1									
K. pneumoniae with NDM (*n*=95)	MEM alone							1		1	3	10	80
	MEM-ZN148	16	30	14	4	15	11	2	2	1			
K. pneumoniae with VIM (*n*=17)	MEM alone							2	2	1	4	3	5
	MEM-ZN148	8	5	2	1		1						
Other *Enterobacterales* with MBLs (*n*=10)	MEM alone								1	2	3	2	2
	MEM-ZN148	1	4	2	3								
All (*n*=234)	MEM alone					1	2	3	10	9	22	35	152
	MEM-ZN148	62	73	45	19	16	13	2	2	2			

[a] Includes one *E. coli* isolate co-producing NDM-1 and VIM-1

Table S3.

MICs of meropenem (MEM), doripenem (DOR) and imipenem (IPM) alone and in combination with 50µM ZN148, against NDM-1-producing *A. baumannii* strains (*n*=6).

Species	MBL	MIC (mg/L)					
		MEM	MEM-ZN148	DOR	DOR-ZN148	IPM	IPM-ZN148
A. baumannii	NDM-1	>64	32	>64	32	>64	8
A. baumannii	NDM-1	>64	16	>64	16	>64	8
A. baumannii	NDM-1	>64	4	>64	4	>64	4
A. baumannii	NDM-1	>64	64	>64	64	>64	32
A. baumannii	NDM-1	>64	4	>64	8	>64	8
A. baumannii	NDM-1	>64	8	>64	4	>64	8

Table S4.
MICs of meropenem (MEM), doripenem (DOR) and imipenem (IPM) alone and in combination with 50μM ZN148, against strains co-producing MBLs and class A/D serine carbapenemases (*n*=38).

Species	MBL	Class A/D carbapenemase	MIC (mg/L)					
			MEM	MEM-ZN148	DOR	DOR-ZN148	IPM	IPM-ZN148
E. coli	NDM-1	OXA-181	4	0.5	2	0.25	4	2
E. coli	NDM-1	OXA-181	32	0.25	32	0.25	16	2
E. coli	NDM-1	OXA-181	>64	2	>64	4	>64	8
E. coli	NDM-5	OXA-181	64	0.5	64	0.25	16	0.5
E. coli	NDM-5	OXA-181	64	0.5	64	0.25	16	1
E. coli	NDM-5	OXA-181	64	0.5	64	0.25	16	2
E. coli	NDM-5	OXA-232	>64	2	>64	0.5	>64	1
E. coli	NDM-5	OXA-181	>64	1	64	0.25	32	0.25
E. coli	NDM-5	OXA-48	>64	2	>64	1	32	8
E. coli	NDM-1	GES-2	>64	0.06	64	0.03	16	0.12
E. coli	NDM-5	OXA-232	>64	1	>64	0.5	64	1
K. pneumoniae	NDM-1	OXA-232	>64	64	>64	64	>64	16
K. pneumoniae	NDM-1	OXA-232	>64	32	>64	32	>64	8
K. pneumoniae	NDM-1	OXA-232	>64	64	>64	32	>64	16
K. pneumoniae	NDM-1	OXA-232	>64	32	>64	16	>64	8
K. pneumoniae	NDM-1	OXA-232	>64	16	>64	16	>64	4
K. pneumoniae	NDM-1	OXA-232	>64	16	>64	8	>64	8
K. pneumoniae	NDM-1	OXA-232	>64	32	>64	32	>64	8
K. pneumoniae	NDM-1	OXA-232	>64	16	>64	16	>64	4
K. pneumoniae	NDM-1	OXA-232	>64	32	>64	16	>64	4
K. pneumoniae	NDM-1	OXA-232	>64	16	>64	8	>64	**2**
K. pneumoniae	NDM-5	OXA-181	>64	32	>64	32	>64	16
K. pneumoniae	NDM-1	OXA-232	>64	32	>64	32	>64	4
K. pneumoniae	NDM-1	OXA-232	>64	16	>64	16	>64	4
K. pneumoniae	NDM-1	OXA-232	>64	32	>64	16	>64	16
K. pneumoniae	NDM-5	OXA-181	>64	32	>64	16	>64	8
K. pneumoniae	NDM	OXA-48-like	>64	32	ND[a]	ND	ND	ND
K. pneumoniae	NDM	OXA-48-like	>64	32	ND	ND	ND	ND
K. pneumoniae	NDM-1	OXA-181	>64	64	ND	ND	ND	ND
K. pneumoniae	NDM-1	OXA-232	64	16	ND	ND	ND	ND
M. morganii	NDM-1	OXA-48	8	1	ND	ND	ND	ND
A. baumannii	NDM-1	OXA-24-like; OXA-58-like	>64	>64	>64	>64	>64	64
A. baumannii	NDM-1	OXA-24-like; OXA-58-like	>64	>64	>64	>64	64	64
A. baumannii	NDM-1	OXA-23-like	>64	64	>64	64	>64	64
A. baumannii	NDM-1	OXA-23-like	>64	64	>64	64	>64	32
A. baumannii	NDM-1	OXA-23-like	>64	64	>64	64	>64	32
A. baumannii	NDM-1	OXA-23-like	>64	64	>64	64	>64	32
A. baumannii	NDM-1	OXA-23-like	>64	32	>64	64	>64	64

[a] ND: not determined.

Table S5.

Minimum inhibitory concentrations (MICs) of meropenem (MEM), doripenem (DOR) and imipenem (IPM) alone and in combination with 50µM ZN148, against class A carbapenemase-producing strains (*n*=10).

Species	Class A carbapenemase	MIC (mg/L)					
		MEM	MEM-ZN148	DOR	DOR-ZN148	IPM	IPM-ZN148
E. coli	KPC-2	8	8	4	4	16	8
E. coli	KPC-2	4	4	2	2	4	4
E. coli	KPC-3	2	2	4	4	8	8
E. cloacae	KPC-2	16	8	4	4	8	8
E. cloacae	KPC-2	32	32	16	8	16	16
E. cloacae	KPC-3	2	2	2	2	16	4
K. pneumoniae	KPC-2	>64	>64	>64	>64	>64	64
K. pneumoniae	KPC-3	2	2	1	1	4	4
K. pneumoniae	KPC-2	2	2	2	1	4	4
K. pneumoniae	KPC-3	64	64	16	16	32	16

Table S6.
Resistance frequency of *K. pneumoniae* K66-45 to ZN148-meropenem co-treatment, determined by single-step selection on agar. MIC of meropenem alone: >32 mg/L. MIC of meropenem in presence of 30 µM, 50 µM or 100 µM ZN148: 0.125 mg/L.

ZN148 (µM)	Frequency of resistance				
	Meropenem (mg/L)				
	0.5 (S[a])	1 (S[a])	2 (S[a])	4 (I[b])	8 (I[b])
30	2.7 x10^{-7}	1.4 x10^{-7}	1.2 x10^{-7}	4.0 x10^{-9}	n.c.[c]
50	2.2 x10^{-7}	7.4 x10^{-8}	3.5 x10^{-8}	n.c.	n.c.
100	3.0 x10^{-7}	2.2 x10^{-8}	n.c.	n.c.	n.c.
200	2.7 x10^{-7}	n.d.[d]	n.d.	n.d.	n.d.

[a] S: sensitive; [b] I: intermediate; [c] n.c.: no colonies; [d] n.d.: not determined.

Table S7.
Variants identified in spontaneous ZN148-meropenem resistant mutants. Variant frequency was >97% in all mutants.

ZN148 (µM) + meropenem (mg/L)	Product(s)	Protein effect	Amino acid change	Distribution
50+2	OmpK36	Substitution	L > Q	3/9
50+2	OmpK36	Substitution	T > P	1/9
50+2	OmpK36	Frame shift	-	1/9
50+2	OmpK36	Truncation	-	1/9
50+2	No change	-	-	3/9
100+1	LTTR[a]	Substitution	F > L	4/7
100+1	LTTR[a]; OmpK36	Substitution; truncation	F > L; -	2/7
100+1	No change	-	-	1/7

[a] LTTR: LysR family transcriptional regulator

Table S8.
High frequency variants identified in ZN148-meropenem resistant mutants isolated from serial passaging in the presence of 100 µM ZN148 and 2 mg/L meropenem.

Product(s)	Protein effect	Amino acid change	Distribution
OmpK36	Substitution	R -> L	1/6
OmpK36	Truncation	-	1/6
LysR family transcriptional regulator; OmpK36	Substitution; truncation	F > L; -	1/6
